# Descriptor-Driven Prediction of Adsorption Energy of Oxygenates on Metal Dioxide Surfaces

**DOI:** 10.1021/acs.jpcc.5c00005

**Published:** 2025-03-25

**Authors:** Chen Chen, Zhihui Li, Jia Yang, Haifeng Wang, De Chen

**Affiliations:** † Key Laboratory for Advanced Materials, Centre for Computational Chemistry and Research Institute of Industrial Catalysis, East China University of Science and Technology, Shanghai 200237, China; ‡ Department of Chemical Engineering, Norwegian University of Science and Technology, Trondheim 7034, Norway; § The College of Smart Energy, Shanghai Jiao Tong University, Shanghai 200237, China

## Abstract

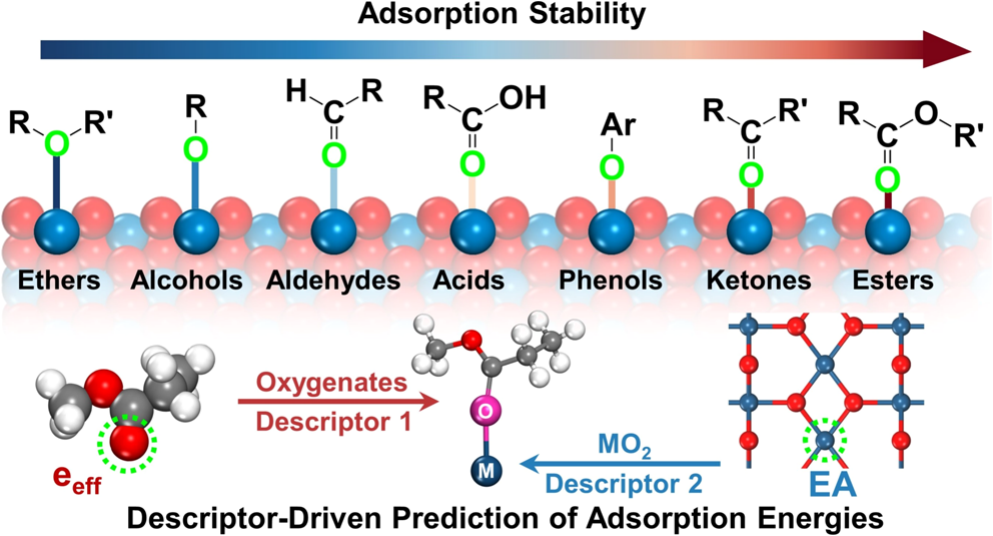

Adsorption is a critical factor in heterogeneous catalysis, as the interaction between adsorbate and adsorbent significantly impacts catalytic efficiency and selectivity. In this study, we utilized density functional theory (DFT) to comprehensively analyze the adsorption behavior of various oxygenates on the surfaces of metal dioxide (MO_2_) catalysts. Our findings reveal a strong dependence of adsorption energy (*E*_ad_) on two primary descriptors: the effective charge (*e*_eff_) of oxygen atoms in oxygenates and the electron affinity (EA) of the surface metal atoms in MO_2_. We observed that oxygenates with more negative *e*_eff_ exhibit stronger adsorption, while MO_2_ with lower EA offer greater adsorption stability. Using these two descriptors, a predictive *E*_ad_ scaling relationship was developed and validated across different MO_2_ surfaces. This descriptor-based model establishes an efficient framework for accurately predicting adsorption strength and offers valuable theoretical insights for designing and screening MO_2_ catalysts with optimized adsorption properties.

## 1 Introduction

Metal oxides are essential catalysts for converting oxygenates and facilitating the sustainable transformation of feedstocks into valuable chemicals and fuels.^1−4^ Their high thermal stability allows them to endure rigorous reaction conditions, supporting efficient and reusable catalysis.^5−8^ Among these, metal dioxides (MO_2_) exhibit tunable acid–base and redox properties that drive selective reactions, enhancing yield and reducing byproducts.^9,10^ Redox-active MO_2_, such as iridium dioxide (IrO_2_), titanium dioxide (TiO_2_), tin dioxide (SnO_2_), and cerium dioxide (CeO_2_), facilitate crucial electron transfer, thereby accelerating reaction rates.^11−16^ These attributes underscore the significant potential of MO_2_ in catalytic applications.^17−19^

The adsorption of oxygenates on MO_2_ surfaces is crucial for understanding reaction mechanisms, controlling selectivity, and preventing catalyst deactivation. The redox properties of MO_2_ enable the sustainable conversion of biomass oxygenates into fuels and chemicals. Moreover, adsorption determines active site distribution and reaction pathways, and understanding the adsorption properties is key to tuning catalysts and improving performance.^20−22^ In particular, during the conversion of oxygenates (such as alcohols, aldehydes, ketones, and acids), adsorption influences the kinetics of catalytic reactions, thereby having a major impact on reaction rates and efficiency.^23−26^ For example, Mallesham et al. significantly enhanced the adsorption of acetone on sulfate-modified SnO_2_ surfaces, accelerating the acetalization reaction and achieving a conversion rate of up to 99%.^27^ Research by Tsukamoto et al. demonstrated that inhibiting the adsorption of aldehydes on TiO_2_ surfaces is an effective strategy for increasing the selectivity of alcohol oxidation, promoting the conversion of alcohols to aldehydes.^28^ Additionally, Wei et al. discovered that the stable adsorption of aldehydes on the CeO_2_–ZrO_2_ support is the key factor underlying its exceptional catalytic hydrogenation performance.^29^ Therefore, a thorough understanding of the adsorption mechanisms of oxygenates on MO_2_ surfaces is crucial for rationally tuning the reactivity of adsorption sites and maximizing catalytic performance.

Current experimental and computational methods provide insights into adsorption on catalyst surfaces, but they are often complex and computationally expensive.^30−34^ Addressing these challenges, Nørskov et al. introduced descriptors such as electrostatic interactions, density of states, and d-electrons to predict adsorption and catalytic activity on metal surfaces.^35^ Calle-Vallejo et al. highlighted the outer electron count as a predictor of adsorption energy (*E*_ad_) for reaction intermediates on transition metals and their oxides.^36^ Although these approaches offer promising strategies for predicting adsorption activity, studies of oxygenates adsorption on MO_2_ remain limited, which restricting the optimizing and design of MO_2_ catalytic.

In this study, we systematically investigated the adsorption behavior of seven oxygenates—alcohols, aldehydes, ketones, acids, ethers, esters, and phenols—on the surfaces of various MO_2_ catalysts, including IrO_2_, SnO_2_, TiO_2_, PtO_2_, CeO_2_, and ZrO_2_, using density functional theory (DFT). Our findings reveal that the effective charge (*e*_eff_) of oxygen atoms in oxygenates and the electron affinity (EA) of MO_2_ are key descriptors for predicting *E*_ad_. Based on these, we developed a new formula for calculating *E*_ad_ and validated its accuracy on five additional MO_2_ (VO_2_, PdO_2_, TaO_2_, HfO_2_, and NbO_2_) catalyst surfaces. This work enhances our understanding of adsorption mechanisms, reduces computational and experimental burdens, and provides theoretical guidance for efficient catalyst design and screening.

## 2 Computation Methods

The simulations were performed with the Vienna ab initio simulation package (VASP).^37,38^ All density functional theory (DFT) calculations were conducted using the Perdew–Burke–Ernzerhof (PBE) functional within the generalized gradient approximation (GGA).^39^ To account for core–valence electron interactions, the projector-augmented wave (PAW) method was employed. Valence electronic states were described using plane wave basis sets with an energy cutoff of 450 eV. Structural optimization was completed when the maximum forces on relaxed atoms fell below 0.05 eV/Å.^40^ A 1 × 1 × 1 *k*-point mesh was applied because of the large supercell dimensions (30 × 30 × 30 Å^3^). According to previous reports, the on-site Hubbard *U* term (DFT + *U*) was applied to the Ir 5d, Sn 5s, Ti 3d, Pt 5d, Zr 4d, Ce 4f, Hf 5d, Nb 4d, and Ta 5d orbitals, with *U* values of 2, 3.5, 4.2, 4, 5, 5, 6, 2, and 2 eV, respectively.^41−49^ For a more detailed explanation regarding the selection of the *U* value, please refer to Supporting Note 1.1 in Supporting Information.

The formula for calculating adsorption energy (*E*_ad_) in the gas phase is given by *E*_ad_ = *E*_X/surf_ – *E*_surf_ – *E*_X_ + ΔZPE + *T*Δ*S*.^50^ In this expression, *E*_X/surf_ is the total energy of the adsorbed molecule X on the surface; *E*_surf_ is the total energy of the surface without the adsorbed molecule; and *E*_X_ is the total energy of the molecule X in the gas phase. ΔZPE = ZPE_X/surf_ – ZPE_surf_ – ZPE_X_, where ZPE_X/surf_, ZPE_surf_ and ZPE_X_ represent the zero-point energy of the adsorbed molecule on the surface, the bare surface, and the molecule in the gas phase, respectively. *T* is the temperature (typically 298 K, room temperature). Δ*S* = *S*_X/surf_ – *S*_X_, where Δ*S* is the change in entropy before and after adsorption; *S*_X/surf_ is the entropy of the molecule in the adsorbed state and *S*_X_ is the entropy of the molecule in the gas phase. The specific effects of Δ*S* and ΔZPE are discussed in Supporting Note 1.2 in the Supporting Information. After performing static calculations with VASP, vibrational frequency analysis is conducted. VASPKIT is used to extract entropy and zero-point energy information for correction, and this method has been validated for its accuracy.^51^ The exchange-correlation interactions were described using the vdW-DF method, which combines a nonlocal correlation functional with a consistent exchange functional to account for van der Waals forces accurately.^31,52^ To more accurately describe the strength of chemical bonds, bond energies are calculated using the Integrated Crystal Orbital Hamilton Population (ICOHP) method.^53^ All calculations are performed using the Lobster program in conjunction with VASP.^54,55^ Additionally, the electron affinity (EA) is calculated using the formula EA = *E*_neutral_*– E*_charged_, where *E*_neutral_ is the total energy of the neutral system, and *E*_charged_ is the total energy of the negatively charged system after introducing an extra electron and structural relaxation. This difference reflects the EA of the system. The above DFT setup affords good accuracy, proved by previous work.^56,57^

## 3 Results and Discussion

### 3.1 Structure of MO_2_ Surfaces and Oxygenates

Based on their superior catalytic performance and widespread application in biomass conversion reactions, we selected IrO_2_, SnO_2_, TiO_2_, PtO_2_, CeO_2_, and ZrO_2_ as the MO_2_ model adsorbents.^58,59^ We also chose widely studied active crystal phases and facets from the literature, including rutile-IrO_2_(110), rutile-SnO_2_(110), rutile-TiO_2_(110), tetragonal-PtO_2_(111), tetragonal-ZrO_2_(111), and tetragonal-CeO_2_(111),^60−65^ to investigate the adsorption stability of oxygenates on the surfaces of MO_2_.

**Figure 1:**
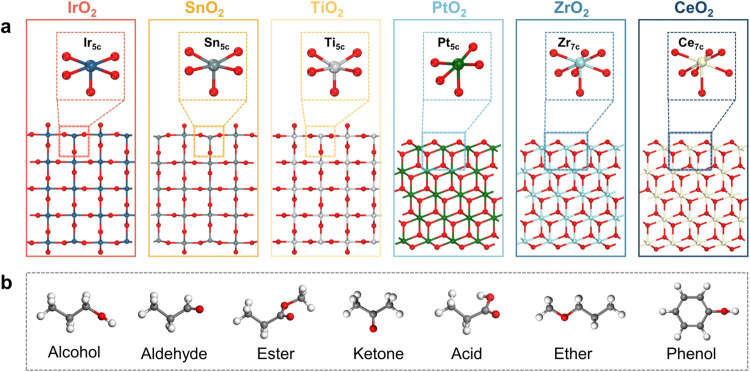
(a) Structure of various MO_2_ surfaces, including rutile-IrO_2_(110), rutile-SnO_2_(110), rutile-TiO_2_(110), tetragonal-PtO_2_(111), tetragonal-ZrO_2_(111), and tetragonal-CeO_2_(111). Insets show the local coordination environments of the metal sites. (b) Molecular structures of oxygenates, including C3-alcohol, C3-aldehyde, methyl propionate (ester), C3-ketone, C3-acid, propyl methyl ether, and phenol. Color scheme: dark blue (Ir), light green (Sn), light gray (Ti), green (Pt), light blue (Zr), light yellow (Ce), red (O), gray (C), and white (H). This color scheme is used throughout the paper.

As shown in Figure 1a, IrO_2_(110), SnO_2_(110), and TiO_2_(110) surfaces are exhibit a terrace-step structure, exposing two-coordinated bridging oxygen sites (O_2c_) and five-coordinated metal sites (M_5c_). Additionally, the PtO_2_(111), CeO_2_(111), and ZrO_2_(111) surfaces are characterized by a zigzag structure. Moreover, CeO_2_(111) and ZrO_2_(111) surfaces exposes three-coordinated oxygen sites (O_3c_) and seven-coordinated metal sites (M_7c_). The main active centers of the adsorption reaction are the metal sites, and the adsorption behavior depends to a large extent on the geometry and electronic structure of these sites. The subsequent results and discussion in this study are based on these extensively studied MO_2_ surfaces, especially the terrace-step or zigzag active surfaces of rutile and tetragonal phases. To systematically study the adsorption behavior of oxygenates on the MO_2_ surface, we selected seven representative categories of compounds, including alcohols, aldehydes, ketones, acids, ethers, esters, and phenols. Using different categories of C3 molecules (e.g., propanol, acetone, propionic acid, etc., excluding phenol) as model compounds to analyze their adsorption stability and interaction mechanisms on the surfaces of MO_2_ (Figure 1b).

### 3.2 Adsorption Energy

**Figure 2:**
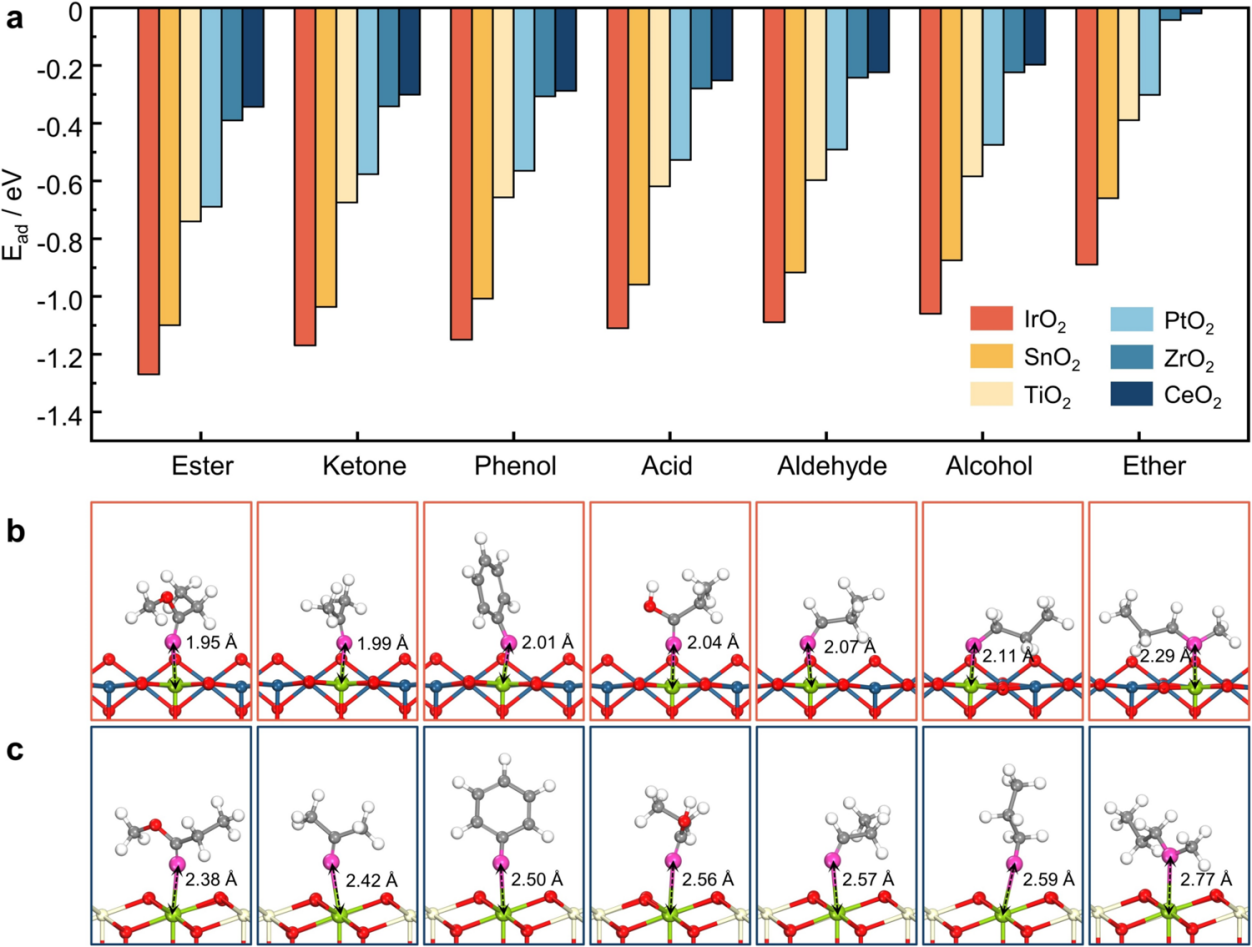
(a) *E*_ad_ of oxygenates on various MO_2_ surfaces, including rutile-IrO_2_(110), rutile-SnO_2_(110), rutile-TiO_2_(110), tetragonal-PtO_2_(111), tetragonal-ZrO_2_(111), and tetragonal-CeO_2_(111). (b, c) Adsorption structures of oxygenates on the rutile-IrO_2_(110) and tetragonal-CeO_2_(111) surfaces. Green highlights the Ir and Ce adsorption sites, while pink indicates the adsorbed oxygen atoms. Black markers denote bond lengths.

The adsorption behavior of seven oxygenates on six different MO_2_ surfaces was investigated, and the corresponding adsorption energies (*E*_ad_) and configurations are presented in Figures 2a–c and S4. Overall, the interactions are primarily governed by the bonding between oxygen atoms in the oxygenates and the exposed metal sites on the MO_2_ surfaces.^66^ DFT calculations indicate that oxygenates bearing hydroxyl (−OH) groups, such as alcohols and phenols, generally undergo dissociative adsorption, consistent with earlier reports in the literature.^26,67,68^ By contrast, acids preferentially adsorb via the C=O bond, which proves energetically more favorable than the dissociative pathway (−OH dehydrogenation). For instance, on the IrO_2_ surface, *E*_ad_ of the acid via the C=O group is −1.11 eV, while −OH dehydrogenation (−O) adsorption is only exothermic by 0.73 eV. Therefore, the most stable C=O-bonded structure was used for subsequent discussions on acid adsorption.

**Figure 3:**
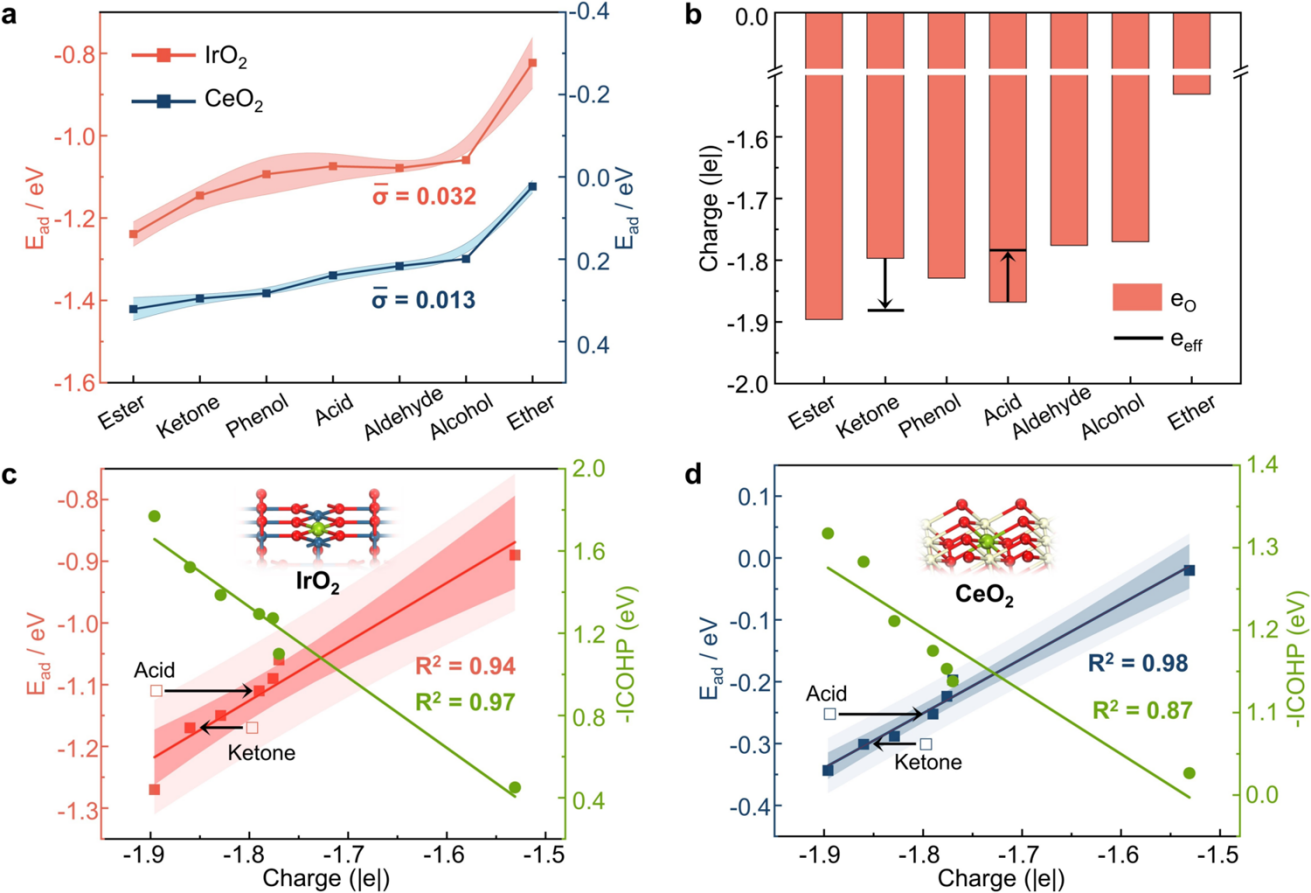
(a) Error bar chart of *E*_ad_ of oxygenates with varying carbon chain lengths (C3, C4, and C6) on rutile-IrO_2_(110) and tetragonal-CeO_2_(111) surfaces. In the case of phenol, the C3, C4, and C6 designations refer to the addition of alkyl groups (−C_3_H_7_, −C_4_H_9_, and −C_6_H_13_) to the benzene ring, corresponding to phenol derivatives with different carbon chain lengths. (b) Bader charges of oxygen atoms (*e*_O_) in oxygenates, and the black markers represent the effective charge (*e*_eff_) of oxygen atoms in oxygenates. (c, d) Correlation between the *e*_eff_ and *E*_ad_ on rutile-IrO_2_(110) and tetragonal-CeO_2_(111) surfaces, along with M–O bond energies (-ICOHP). The M–O bond refers to the bond formed between the metal site of MO_2_ and the oxygen atom of the adsorbed oxygenates. The red and blue lines represent the best-fit regression models for *E*_ad_ on rutile-IrO_2_(110) and tetragonal-CeO_2_(111) surfaces, respectively. The green line shows the correlation between the *e*_eff_ and M–O bond energies. The shaded regions around the lines represent the 95% confidence intervals, indicating the uncertainty in the predicted mean values. The outer shaded regions represent the 95% prediction intervals, showing the expected range of future observations with 95% confidence.

As shown in Figures 3a and S5, we compared the *E*_ad_ values for oxygenates with varying carbon chain lengths (C3, C4, and C6) on MO_2_ surfaces. Tables S9–S14 present the *E*_ad_ of oxygenates with C3, C4, and C6. In all cases, the standard deviations are below 0.032. Therefore, at least within this range of C3–C6 linear molecules, the effect of steric hindrance is minimal, and variations in carbon chain length have a negligible impact on *E*_ad_. These findings are consistent with previous reports.^69,70^ Accordingly, we chose C3-based molecules as representative adsorbates to optimize computational efficiency without compromising accuracy.

Moreover, our *E*_ad_ results align well with prior studies. For instance, Giuliano et al. reported an *E*_ad_ of 0.60 eV for alcohols on TiO_2_, matching closely with our calculated value of 0.58 eV.^69^ Similarly, Alfredo et al. obtained an *E*_ad_ of 0.33 eV for phenol on CeO_2_, compared with our result of 0.29 eV.^71^ The computed adsorption configuration was also in good agreement, featuring a Ce–OC_6_H_6_ bond length of about 2.5 Å. These comparisons confirm the reliability of our DFT results.

As shown in Figure 2a, a clear trend in *E*_ad_ is observed across different adsorbates, in the order: esters > ketones > phenols > acids > aldehydes > alcohols > ethers. The relatively sparse data points in the range of −1.55|e| to −1.75|e| stem from the selection of commonly studied oxygenates, which do not naturally occupy this charge region. Moreover, the bonding between the double-bonded oxygen in esters and ketones and the metal sites enhances the exothermic nature of the adsorption process, leading to greater adsorption stability. According to reports by Balajka et al., the adsorption of oxygenates containing double-bonded oxygen is more stable, which aligns with the conclusions of our study.^70^ For instance, on IrO_2_ surfaces, the strongest adsorption is observed for methyl propionate, with an *E*_ad_ of −1.27 eV, corresponding to an Ir–O bond energy of 1.77 eV and a bond length of 1.95 Å. In contrast, for the weakest adsorption—methyl propyl ether—the *E*_ad_ is only −0.89 eV, with an Ir–O bond energy of 0.45 eV and a bond length of 2.29 Å. This variation in adsorption strength reflects the differing interactions between adsorbates and metal sites. The *E*_ad_ of oxygenates on the surfaces of MO_2_ are presented in Tables S15–S17. Similar adsorption trends were observed across the five catalyst surfaces, demonstrating a high level of consistency in adsorption stability.

### 3.3 Dependence of Adsorption Energy on the Oxygenate Properties

The essence of adsorption bonding lies in electron transfer and electron cloud overlap, making the electronic properties of the adsorbate crucial for adsorption stability. To investigate how the molecular properties of oxygenates affect adsorption strength, various properties, such as C–O and C–H bond energy and bond length, as well as the charge on oxygen and carbon atoms, were estimated and correlated with *E*_ad_. As shown in Figure 3b, we performed a quantitative analysis of the oxygen atom charge in oxygenates using the widely applied Bader charge analysis.^72−75^ The results indicate significant differences in the charge of the oxygen atoms across different molecules. Notably, oxygen atoms in ester molecules carry the most negative charge, while those in ether molecules carry the least negative charge. This distinction arises from their electronic structures: in esters, the carbonyl double bond increases oxygen’s electronegativity and enhances the π-electron delocalization effect, shifting the electron cloud toward the oxygen atom and reducing the availability of lone pair electrons for sharing. Conversely, in ether compounds (e.g., R–O–R’), the oxygen atom is bonded to two carbon atoms via single bonds, resulting in a weaker electron-withdrawing effect and a more uniform electron distribution, which gives the oxygen atom a more positive charge.

Among the different molecule properties studied, a correlation between the *E*_ad_ and the Bader charge of the oxygen atom (*e*_O_) in these molecules was observed. However, using IrO_2_ as an example, directly correlating *e*_O_ with the *E*_ad_ yields a relatively weak fit (*R*^2^ = 0.80), primarily due to deviations from acids and ketones. Moreover, this weakened correlation resulting from acids and ketones is consistently observed across all investigated MO_2_ surfaces. This phenomenon can be attributed to the polarization and conjugation effects of acids and ketones. Specifically, in acid molecules, the high polarity of the carboxyl oxygen induces electron cloud migration toward the carbonyl (C=O) double bond, leading to a higher negative charge on the oxygen atom. In contrast, the conjugation effect in ketone molecules delocalizes the electron density of the oxygen atom, reducing its local negative charge and making the oxygen atomic charge more positive.^76,77^ However, the Bader charge analysis method cannot fully capture these intricate electronic effects, thereby affecting the accuracy of the correlation.

To address this limitation, we introduced an empirical correction to the descriptor, namely the oxygen atom’s charge for oxygenate molecules in acids and ketones.^78,79^ Notably, these correction factors are intrinsic molecular properties and therefore remain constant across different metal dioxide surfaces. The corrected effective charge (*e*_eff_) is expressed as
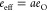
1where *e*_O_ is the original oxygen atom charge and *a* is the correction factor. For acid and ketone molecules, the correction factors are 1.05 and 0.95, respectively.

The *e*_eff_ of oxygen atoms in oxygenates are shown in Table S18. These corrections effectively account for polarization and conjugation effects, yielding corrected oxygen charges of −1.86|e| for acids and −1.79|e| for ketones. As shown in Figures 3c,d and S6, applying these corrections significantly improves the linear correlation between *e*_eff_ and the *E*_ad_ with an *R*^2^ value exceeding 0.94 (IrO_2_). Furthermore, the strong correlation between *e*_eff_ and M–O bond energy confirms the descriptor’s reliability. The more negative the corrected oxygen charge, the stronger the electron transfer, leading to enhanced adsorption interactions with metal sites. These results demonstrate the robustness of the empirical correction method and its applicability across various MO_2_ surfaces.

**Figure 4:**
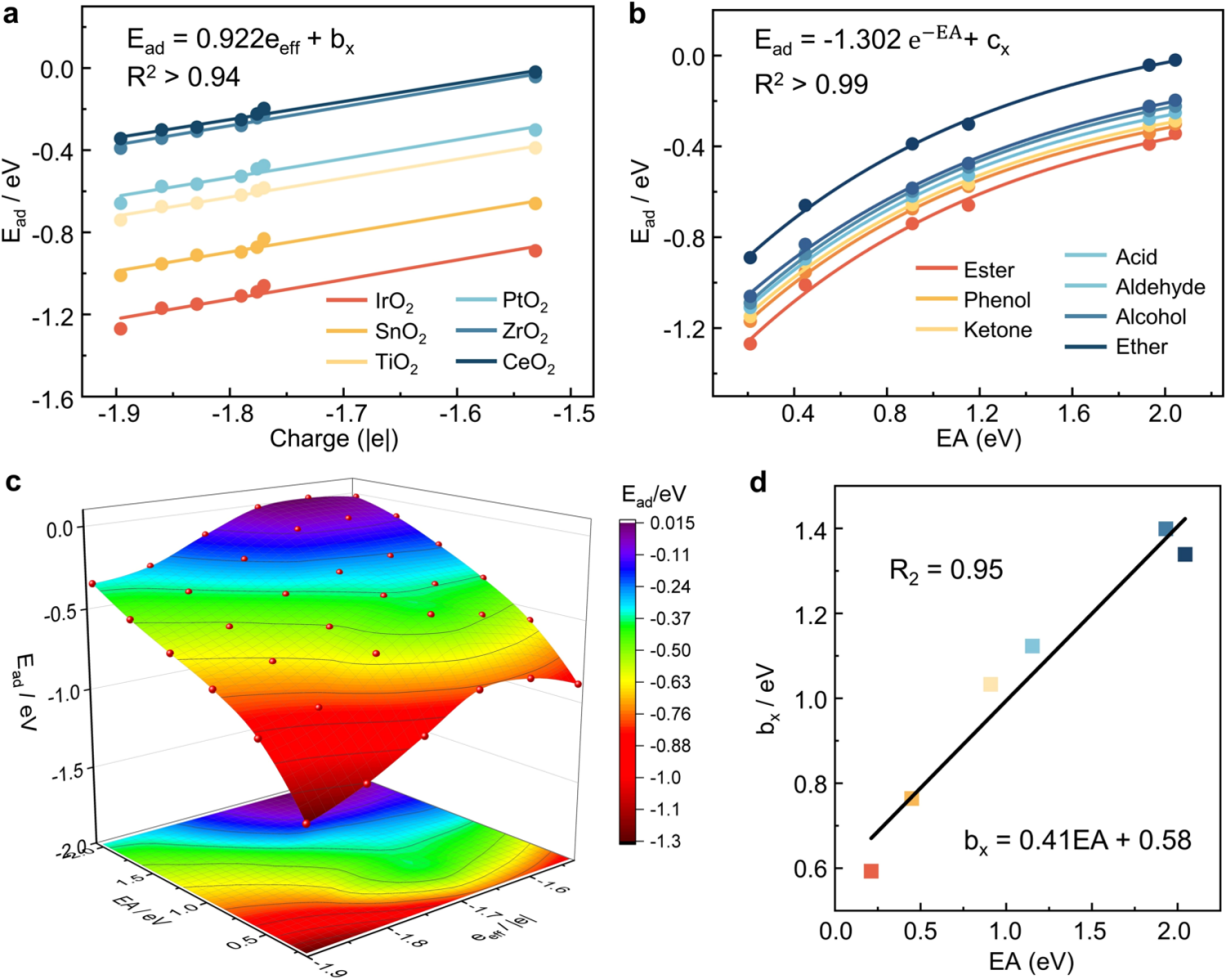
(a) Correlation between the *E*_ad_ and the *e*_eff_. (b) Correlation between the EA and the *E*_ad_. (c) Three-dimensional plot illustrating the relationship between *E*_ad_ and the two descriptors (*e*_eff_ and EA). (d) The linear fit between *b*_*x*_ and EA.

As shown in Figure 4a, the fitting lines for *E*_ad_ and *e*_eff_ across different MO_2_ surfaces are nearly parallel, with an average slope of 0.922. This results in the following expression for *E*_ad_
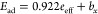
2Where *b*_*x*_ is the fitting constant reflecting the intrinsic properties of MO_2_. The values of *k_x_* (average slope), *b*_*x*_ (intercept), and *R*^2^ (goodness-of-fit) are shown in Table S19. This linear relationship implies that for a given MO_2_ surface, once the constant *b*_*x*_ is determined, the *E*_ad_ of oxygenates can be predicted based on *e*_eff_. Thus, *e*_eff_ serves as an effective descriptor of *E*_ad_, providing a theoretical basis for predicting and controlling adsorption behavior on MO_2_ surfaces.

### 3.4 Dependence of Adsorption Energy on the Metal Dioxides

The *E*_ad_ of oxygenates on MO_2_ depends not only on the properties of adsorbate but also closely relates to the properties of the adsorbent. The *E*_ad_ are shown in Table S15. The same adsorbate exhibits a consistent adsorption trend on different MO_2_ surfaces: IrO_2_ > SnO_2_ > TiO_2_ > PtO_2_ > ZrO_2_ > CeO_2_. This phenomenon is determined by the geometric and electronic structure of the adsorption active sites on the catalyst surface. First, the platform-step structured surfaces (rutile-IrO_2_(110), rutile-SnO_2_(110), rutile-TiO_2_(110)) exhibit better adsorption stability for oxygenates compared to the zigzag surfaces (tetragonal-PtO_2_(111), tetragonal-ZrO_2_(111), and tetragonal-CeO_2_(111)). The flat surfaces provide more space for molecular adsorption, while the increased steric hindrance on the zigzag surfaces lead to a decrease in adsorption stability. Additionally, oxygen atoms in oxygenates usually carry a strong negative charge, which facilitates electron transfer to the metal sites on MO_2_ surfaces. Consequently, the ability of MO_2_ to accept electrons significantly influences adsorption stability: the stronger the electron acceptance capability, the more stable the adsorption.

**Figure 5:**
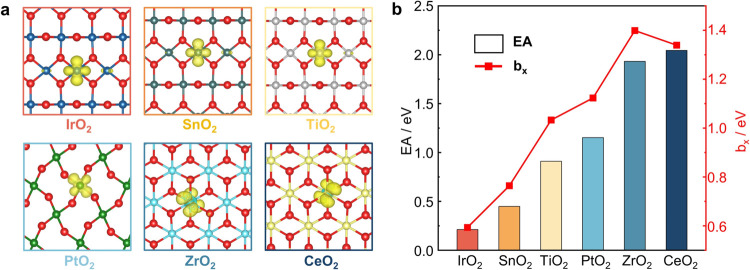
(a) Spin state density plots of an electron localized on the MO_2_ surface. (b) The EA of MO_2_ and the *b*_*x*_ in eq 2.

As shown in Figure S7, we calculated the electron affinity (EA) of metal sites on various MO_2_ surfaces, which shows a certain scaling relationship with the electronegativity of the metal elements. The formula for EA is EA = *E*_neutral_ – *E*_charged_, where *E*_charged_ is the total energy of the system with an additional electron localized at the surface metal site (Figure 5a), and *E*_neutral_ is the total energy of the neutral system. As shown in Figure 4b, the EA shows a strong exponential correlation with *E*_ad_ (*R*^2^ > 0.99), and the lower the EA, the stronger the adsorption stability. Therefore, EA is also a valid descriptor for *E*_ad_. For example, the EA of IrO_2_ is 0.21 eV, requiring less energy for electron transfer, which allows it more easily to accept electrons from the adsorbate, resulting in stronger interactions with oxygenates. In contrast, CeO_2_ has a much higher EA of 2.04 eV, making it more difficult for oxygen atoms to transfer electrons to the Ce sites, leading to relatively weaker adsorption stability with oxygenates. Moreover, the adsorption trends across different MO_2_ surfaces are consistent, with the fitted curves being nearly parallel, and the average slope is −1.302. Therefore, the *E*_ad_ can be expressed as
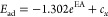
3where *c*_*x*_ is the fitting constant that reflects the properties of the oxygenates (detailed data shown in Table S20).

It is important to note that both EA and the fitting constant *b*_*x*_ characterize the adsorption strength based on the intrinsic properties of MO_2_, and thus they are relevant. As shown in Figure 4d, EA and *b*_*x*_ exhibit consistent trends and demonstrate a strong linear correlation (*R*^2^ = 0.95). Consequently, the unknown coefficient *b*_*x*_ in the *E*_ad_ calculation formula eq 2 can be substituted with the expression for EA
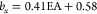
4Combining eqs 2 and 4, *E*_ad_ can be expressed in terms of the properties of the adsorbed molecule (*e*_eff_) and the properties of MO_2_ (EA) as follows in eq 5
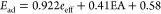
5This equation accounts for the combined effects of oxygenates and surface interactions, providing a more comprehensive explanation of the adsorption mechanism from the perspective of electronic properties. As illustrated in Figure 4c, *E*_ad_ is plotted as a function of two descriptors: one associated with the adsorbate (*e*_eff_) and the other with the adsorbent (EA). Although *e*_eff_ and EA are derived from DFT calculations, they represent intrinsic material properties that remain consistent for a given system. Table S18 presents the *e*_eff_ values for seven oxygenate molecules and the EA values for 11 MO_2_. Due to their intrinsic nature, these parameters can be broadly applied to various adsorption scenarios without necessitating repeated adsorption energy calculations. In contrast to traditional adsorption simulations, which require full DFT optimizations for each adsorption configuration, our approach substantially reduces the computational cost and effort.

### 3.5 Validation

**Figure 6:**
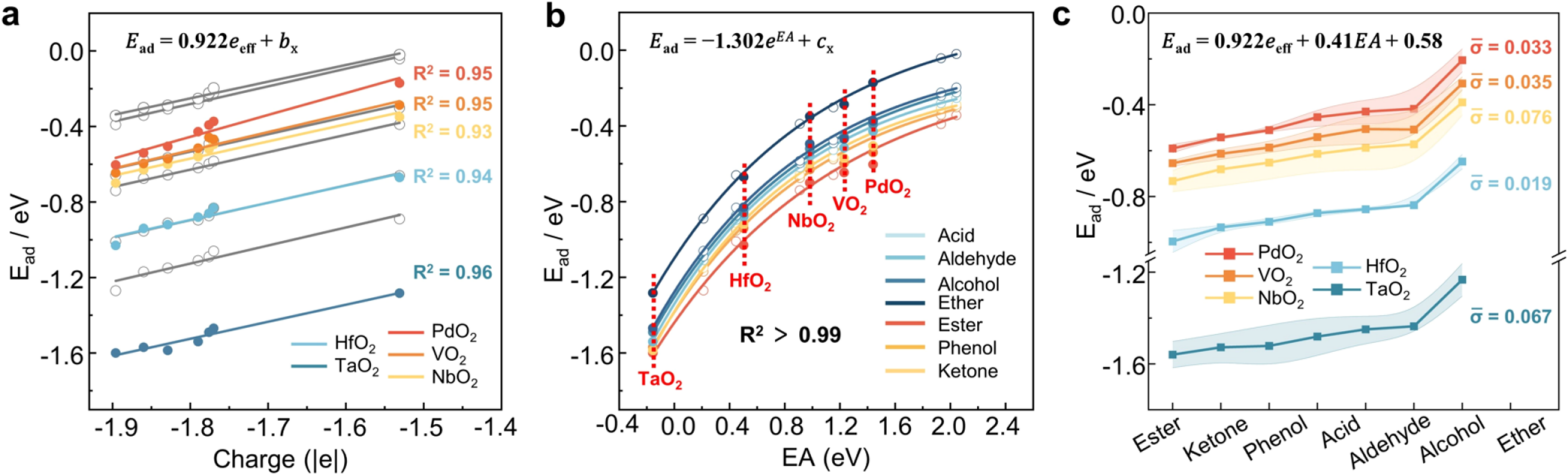
(a) Correlation between the *E*_ad_ of oxygenates on rutile-PdO_2_(100), rutile-VO_2_(100), rutile-TaO_2_(100), tetragonal-HfO_2_(−101) and rutile-NbO_2_(100) and the *e*_eff_ (eq 2). The gray fitting line represents the six MO_2_ previously calculated. (b) Correlation between the EA of rutile-PdO_2_(100), rutile-VO_2_(100), rutile-TaO_2_(100), tetragonal-HfO_2_(−101) and rutile-NbO_2_(100) and the *E*_ad_ of oxygenates on their surface (eq 3). Hollow spheres represent previous calculations and solid spheres represent predictions. (c) Error bar chart of *E*_ad_ error obtained from DFT and prediction eqs (eq 5).

To further validate the accuracy and universality of the proposed *E*_ad_ prediction formula based on the *e*_eff_ and EA descriptors, we compared the *E*_ad_ from DFT-calculated *E*_ad_ with scaling relationship based-prediction on another five different MO_2_ surfaces: rutile-PdO_2_(100), rutile-VO_2_(100), rutile-TaO_2_(100), tetragonal-HfO_2_(−101) and rutile-NbO_2_(100) (*E*_ad_ as shown in Table S21). As illustrated in Figure 6a, the *E*_ad_ calculated using eq 2 exhibits a strong correlation with *e*_eff_ (*R*^2^ > 0.92), with a consistent fitting slope (σ = 0.02). Similarly, the *E*_ad_ predicted using eq 3 shows a strong exponential correlation with EA (Figure 6b), with fitting coefficients comparable to those obtained for previously analyzed MO_2_ systems (σ = 0.04).

Overall, the *E*_ad_ predicted using *e*_eff_ and EA closely match the DFT-calculated values, with a standard error below 0.08 (Figure 6c). This indicates that the proposed predictive model exhibits broad applicability across different MO_2_ surfaces and effectively captures the energy variations during the adsorption process. By validating the model on these five MO_2_ surfaces, we further confirm that *e*_eff_ and EA serve as reliable descriptors for assessing the adsorption stability of oxygenates on different MO_2_ surfaces. This finding provides a solid theoretical foundation for the rational selection of catalysts based on adsorption properties.

## 4 Conclusions

In conclusion, using density functional theory (DFT), we have developed an efficient method for predicting adsorption energy (*E*_ad_) based on descriptors. The effective charge (*e*_eff_) of the oxygen atoms involved in adsorption accurately describes the adsorption stability and interaction strength of various oxygenates, from esters with the strongest adsorption to ethers with the weakest. This phenomenon is attributed to the more negative *e*_eff_ in esters compared to ethers. Another descriptor, electron affinity (EA), also shows a strong exponential correlation with *E*_ad_: lower EA facilitates charge transfer, thus enhancing adsorption stability. Based on the descriptors *e*_eff_ and EA, we propose a scaling relationship for predicting *E*_ad_. The accuracy of this method is further validated by comparing its predictions with results obtained from DFT calculations. This model reduces the demand for extensive computational resources, providing a practical approach for screening and designing metal oxide catalysts with optimized adsorption properties.

## Supporting Information Available

Data, including calculation methods (Note 1), adsorption structures of oxygenates on various metal dioxide surfaces (Figure S4), error bar charts (Figure S5) and correlation plots for adsorption energies with effective charges or electron affinities (Figures S6–S7), five metal dioxide structures for validation (Figure S8), and summary tables of adsorption energies (Tables S9–S15), bond energies (Table S16), bond lengths (Table S17), effective charges or electron affinities (Table S18), proportional relationships of oxygenates on different metal dioxide surfaces (Tables S19–S20), and comparison of predicted and DFT-calculated adsorption energies (Table S21) (PDF)

## References

[ref1] Liu Z.-P.; Gong X.-Q.; Kohanoff J.; Sanchez C.; Hu P. Catalytic Role of Metal Oxides in Gold-Based Catalysts: A First Principles Study of CO Oxidation on TiO_2_ Supported Au. Phys. Rev. Lett. 2003, 91, 266102 10.1103/PhysRevLett.91.266102. 14754070

[ref2] Wang L.; Shi C.; Wang L.; Pan L.; Zhang X.; Zou J.-J. Rational design, synthesis, adsorption principles and applications of metal oxide adsorbents: a review. Nanoscale 2020, 12, 4790–4815. 10.1039/C9NR09274A. 32073021

[ref3] Yuan S.; Duan X.; Liu J.; Ye Y.; Lv F.; Liu T.; Wang Q.; Zhang X. Recent progress on transition metal oxides as advanced materials for energy conversion and storage. Energy Storage Mater. 2021, 42, 317–369. 10.1016/j.ensm.2021.07.007.

[ref4] Ahmad S.; Sohail K.; Chen L.; Xu H.; Din H. U.; Zhou Z. Type-II van der Waals heterostructures of GeC, ZnO and Al_2_SO monolayers for promising optoelectronic and photocatalytic applications. Int. J. Hydrogen Energy 2023, 48, 25354–25365. 10.1016/j.ijhydene.2023.03.268.

[ref5] Lang R.; Du X.; Huang Y.; Jiang X.; Zhang Q.; Guo Y.; Liu K.; Qiao B.; Wang A.; Zhang T. Single-Atom Catalysts Based on the Metal–Oxide Interaction. Chem. Rev. 2020, 120, 11986–12043. 10.1021/acs.chemrev.0c00797. 33112599

[ref6] Li Y.; Zhang Y.; Qian K.; Huang W. Metal–Support Interactions in Metal/Oxide Catalysts and Oxide–Metal Interactions in Oxide/Metal Inverse Catalysts. ACS Catal. 2022, 12, 1268–1287. 10.1021/acscatal.1c04854.

[ref7] Yu K.; Lou L.-L.; Liu S.; Zhou W. Asymmetric Oxygen Vacancies: the Intrinsic Redox Active Sites in Metal Oxide Catalysts. Adv. Sci. 2020, 7, 1901970 10.1002/advs.201901970. PMC6974941 31993288

[ref8] Lee J.; Orilall M. C.; Warren S. C.; Kamperman M.; DiSalvo F. J.; Wiesner U. Direct access to thermally stable and highly crystalline mesoporous transition-metal oxides with uniform pores. Nat. Mater. 2008, 7, 222–228. 10.1038/nmat2111. 18223653

[ref9] Metiu H.; Chrétien S.; Hu Z.; Li B.; Sun X. Chemistry of Lewis Acid–Base Pairs on Oxide Surfaces. J. Phys. Chem. C 2012, 116, 10439–10450. 10.1021/jp301341t.

[ref10] Barteau M. A. Organic Reactions at Well-Defined Oxide Surfaces. Chem. Rev. 1996, 96, 1413–1430. 10.1021/cr950222t. 11848796

[ref11] Sharma S.; Hu Z.; Zhang P.; McFarland E. W.; Metiu H. CO_2_ methanation on Ru-doped ceria. J. Catal. 2011, 278, 297–309. 10.1016/j.jcat.2010.12.015.

[ref12] Liao F.; Yin K.; Ji Y.; Zhu W.; Fan Z.; Li Y.; Zhong J.; Shao M.; Kang Z.; Shao Q. Iridium oxide nanoribbons with metastable monoclinic phase for highly efficient electrocatalytic oxygen evolution. Nat. Commun. 2023, 14, 1248 10.1038/s41467-023-36833-1. 36871002 PMC9985653

[ref13] Xie Q.; Zhang H.; Kang J.; Cheng J.; Zhang Q.; Wang Y. Oxidative Dehydrogenation of Propane to Propylene in the Presence of HCl Catalyzed by CeO_2_ and NiO-Modified CeO_2_ Nanocrystals. ACS Catal. 2018, 8, 4902–4916. 10.1021/acscatal.8b00650.

[ref14] Campbell C. T.; Peden C. H. F. Oxygen Vacancies and Catalysis on Ceria Surfaces. Science 2005, 309, 713–714. 10.1126/science.1113955. 16051777

[ref15] Chen J.; Penschke C.; Alavi A.; Michaelides A. Small polarons and the Janus nature of TiO_2_. Phys. Rev. B 2020, 101, 115402 10.1103/PhysRevB.101.115402.

[ref16] Douthwaite M.; Zhang B.; Iqbal S.; Miedziak P. J.; Bartley J. K.; Willock D. J.; Hutchings G. J. Transfer hydrogenation of methyl levulinate with methanol to gamma valerolactone over Cu-ZrO_2_: A sustainable approach to liquid fuels. Catal. Commun. 2022, 164, 106430 10.1016/j.catcom.2022.106430.

[ref17] Farooq U.; Ahmad T.; Naaz F.; Islam S. u., Review on Metals and Metal Oxides in Sustainable Energy Production: Progress and Perspectives. Energy Fuels 2023, 37, 1577–1632. 10.1021/acs.energyfuels.2c03396.

[ref18] Haider M. H.; Dummer N. F.; Zhang D.; Miedziak P.; Davies T. E.; Taylor S. H.; Willock D. J.; Knight D. W.; Chadwick D.; Hutchings G. J. Rubidium- and caesium-doped silicotungstic acid catalysts supported on alumina for the catalytic dehydration of glycerol to acrolein. J. Catal. 2012, 286, 206–213. 10.1016/j.jcat.2011.11.004.

[ref19] Guadix-Montero S.; Sainna M. A.; Jin J.; Reynolds J.; Forsythe W. G.; Sheldrake G. N.; Willock D.; Sankar M. Ruthenium ion catalysed C–C bond activation in lignin model compounds – towards lignin depolymerisation. Catal. Sci. Technol. 2023, 13, 5912–5923. 10.1039/D3CY00076A. 38013724 PMC10577544

[ref20] Balajka J.; Hines M. A.; DeBenedetti W. J. I.; Komora M.; Pavelec J.; Schmid M.; Diebold U. High-affinity adsorption leads to molecularly ordered interfaces on TiO_2_ in air and solution. Science 2018, 361, 786–789. 10.1126/science.aat6752. 30139869

[ref21] Cho H. S.; Yang J.; Gong X.; Zhang Y.-B.; Momma K.; Weckhuysen B. M.; Deng H.; Kang J. K.; Yaghi O. M.; Terasaki O. Isotherms of individual pores by gas adsorption crystallography. Nat. Chem. 2019, 11, 562–570. 10.1038/s41557-019-0257-2. 31086299

[ref22] Ren Y.; Zhang D.; Suo J.; Cao Y.; Eickemeyer F. T.; Vlachopoulos N.; Zakeeruddin S. M.; Hagfeldt A.; Grätzel M. Hydroxamic acid pre-adsorption raises the efficiency of cosensitized solar cells. Nature 2023, 613, 60–65. 10.1038/s41586-022-05460-z. 36288749

[ref23] Li J.; Xu A.; Li F.; Wang Z.; Zou C.; Gabardo C. M.; Wang Y.; Ozden A.; Xu Y.; Nam D.-H.; et al. Enhanced multi-carbon alcohol electroproduction from CO via modulated hydrogen adsorption. Nat. Commun. 2020, 11, 3685 10.1038/s41467-020-17499-5. 32703956 PMC7378828

[ref24] Lainé J.; Foucaud Y.; Bonilla-Petriciolet A.; Badawi M. Molecular picture of the adsorption of phenol, toluene, carbon dioxide and water on kaolinite basal surfaces. Appl. Surf. Sci. 2022, 585, 152699 10.1016/j.apsusc.2022.152699.

[ref25] Wang T.; Tao L.; Zhu X.; Chen C.; Chen W.; Du S.; Zhou Y.; Zhou B.; Wang D.; Xie C.; et al. Combined anodic and cathodic hydrogen production from aldehyde oxidation and hydrogen evolution reaction. Nat. Catal. 2022, 5, 66–73. 10.1038/s41929-021-00721-y.

[ref26] Wang T.; Sha J.; Sabbe M.; Sautet P.; Pera-Titus M.; Michel C. Identification of active catalysts for the acceptorless dehydrogenation of alcohols to carbonyls. Nat. Commun. 2021, 12, 5100 10.1038/s41467-021-25214-1. 34429417 PMC8385104

[ref27] Mallesham B.; Sudarsanam P.; Reddy B. M. Eco-friendly synthesis of bio-additive fuels from renewable glycerol using nanocrystalline SnO_2_-based solid acids. Catal. Sci. Technol. 2014, 4, 803–813. 10.1039/c3cy00825h.

[ref28] Tsukamoto D.; Ikeda M.; Shiraishi Y.; Hara T.; Ichikuni N.; Tanaka S.; Hirai T. Selective photocatalytic oxidation of alcohols to aldehydes in water by TiO_2_ partially coated with WO_3_. Chem. - Eur. J. 2011, 17, 9816–9824. 10.1002/chem.201100166. 21735494

[ref29] Wei S.; Zhao Y.; Fan G.; Yang L.; Li F. Structure-dependent selective hydrogenation of cinnamaldehyde over high-surface-area CeO_2_-ZrO_2_ composites supported Pt nanoparticles. Chem. Eng. J. 2017, 322, 234–245. 10.1016/j.cej.2017.04.026.

[ref30] Zhao H.; Yang Y.; Shu X.; Wang Y.; Ran Q. Adsorption of organic molecules on mineral surfaces studied by first-principle calculations: A review. Adv. Colloid Interface Sci. 2018, 256, 230–241. 10.1016/j.cis.2018.04.003. 29656761

[ref31] Tillotson M. J.; Brett P. M.; Bennett R. A.; Grau-Crespo R. Adsorption of organic molecules at the TiO_2_(110) surface: The effect of van der Waals interactions. Surf. Sci. 2015, 632, 142–153. 10.1016/j.susc.2014.09.017.

[ref32] Thomas A. G.; Syres K. L. Adsorption of organic molecules on rutile TiO_2_ and anatase TiO_2_ single crystal surfaces. Chem. Soc. Rev. 2012, 41, 4207–4717. 10.1039/c2cs35057b. 22517475

[ref33] Lin X.; Du X.; Wu S.; Zhen S.; Liu W.; Pei C.; Zhang P.; Zhao Z.-J.; Gong J. Machine learning-assisted dual-atom sites design with interpretable descriptors unifying electrocatalytic reactions. Nat. Commun. 2024, 15, 8169 10.1038/s41467-024-52519-8. 39289388 PMC11408493

[ref34] Zhao Z.-J.; Liu S.; Zha S.; Cheng D.; Studt F.; Henkelman G.; Gong J. Theory-guided design of catalytic materials using scaling relationships and reactivity descriptors. Nat. Rev. Mater. 2019, 4, 792–804. 10.1038/s41578-019-0152-x.

[ref35] Nørskov J. K. Theory of adsorption and adsorbate-induced reconstruction. Surf. Sci. 1994, 299–300, 690–705. 10.1016/0039-6028(94)90690-4.

[ref36] Calle-Vallejo F.; Inoglu N. G.; Su H.-Y.; Martínez J. I.; Man I. C.; Koper M. T. M.; Kitchin J. R.; Rossmeisl J. Number of outer electrons as descriptor for adsorption processes on transition metals and their oxides. Chem. Sci. 2013, 4, 1245–1249. 10.1039/c2sc21601a.

[ref37] Kresse G.; Furthmüller J. Efficiency of ab-initio total energy calculations for metals and semiconductors using a plane-wave basis set. Comput. Mater. Sci. 1996, 6, 15–50. 10.1016/0927-0256(96)00008-0. 9984901

[ref38] Kresse G.; Furthmüller J. Efficient iterative schemes for ab initio total-energy calculations using a plane-wave basis set. Phys. Rev. B 1996, 54, 11169 10.1103/PhysRevB.54.11169. 9984901

[ref39] Kresse G.; Joubert D. From ultrasoft pseudopotentials to the projector augmented-wave method. Phys. Rev. B 1999, 59, 1758 10.1103/PhysRevB.59.1758.

[ref40] Dai Y.-H. A perfect example for the BFGS method. Math. Program. 2013, 138, 501–530. 10.1007/s10107-012-0522-2.

[ref41] Chen H.-Y. T.; Tosoni S.; Pacchioni G. Adsorption of Ruthenium Atoms and Clusters on Anatase TiO_2_ and Tetragonal ZrO_2_(101) Surfaces: A Comparative DFT Study. J. Phys. Chem. C 2015, 119, 10856–10868. 10.1021/jp510468f.

[ref42] Shan W.; Luo W. Charge transfer and metal–insulator transition in (CrO_2_)m/(TaO_2_)n superlattices. J. Phys.: Condens. Matter 2022, 34, 385001 10.1088/1361-648X/ac8133. 35835091

[ref43] Seriani N.; Jin Z.; Pompe W.; Ciacchi L. C. Density functional theory study of platinum oxides: From infinite crystals to nanoscopic particles. Phys. Rev. B 2007, 76, 155421 10.1103/PhysRevB.76.155421.

[ref44] Akbar W.; Elahi I.; Nazir S. Development of ferromagnetism and formation energetics in 3d TM-doped SnO_2_: GGA and GGA + U calculations. J. Magn. Magn. Mater. 2020, 511, 166948 10.1016/j.jmmm.2020.166948.

[ref45] Panda S. K.; Bhowal S.; Delin A.; Eriksson O.; Dasgupta I. Effect of spin orbit coupling and Hubbard U on the electronic structure of IrO_2_. Phys. Rev. B 2014, 89, 155102 10.1103/PhysRevB.89.155102.

[ref46] Çiftçi Y.; Ergun A.; Colakoglu K.; Delıgoz E. First principles LDA+U and GGA+*U* study of HfO_2_: Dependence on the effective U parameter. Gazi Univ. J. Sci. 2014, 27, 627–636.

[ref47] Wang D.; Sheng T.; Chen J.; Wang H.-F.; Hu P. Identifying the key obstacle in photocatalytic oxygen evolution on rutile TiO_2_. Nat. Catal. 2018, 1, 291–299. 10.1038/s41929-018-0055-z.

[ref48] Weibin Z.; Weidong W.; Xueming W.; Xinlu C.; Dawei Y.; Changle S.; Liping P.; Yuying W.; Li B. The investigation of NbO_2_ and Nb_2_O_5_ electronic structure by XPS, UPS and first principles methods. Surf. Interface Anal. 2013, 45, 1206–1210. 10.1002/sia.5253.

[ref49] Wang H.-F.; Guo Y.-L.; Lu G.-Z.; Hu P. Maximizing the Localized Relaxation: The Origin of the Outstanding Oxygen Storage Capacity of κ-Ce_2_Zr_2_O_8_. Angew. Chem., Int. Ed. 2009, 48, 8289–8292. 10.1002/anie.200903907. 19787672

[ref50] Yuan H.; Chen J.; Wang H.; Hu P. Activity Trend for Low-Concentration NO Oxidation at Room Temperature on Rutile-Type Metal Oxides. ACS Catal. 2018, 8, 10864–10870. 10.1021/acscatal.8b03045.

[ref51] Wang V.; Xu N.; Liu J.-C.; Tang G.; Geng W.-T. VASPKIT: A user-friendly interface facilitating high-throughput computing and analysis using VASP code. Comput. Phys. Commun. 2021, 267, 108033 10.1016/j.cpc.2021.108033.

[ref52] Berland K.; Cooper V. R.; Lee K.; Schröder E.; Thonhauser T.; Hyldgaard P.; Lundqvist B. I. van der Waals forces in density functional theory: a review of the vdW-DF method. Rep. Prog. Phys. 2015, 78, 066501 10.1088/0034-4885/78/6/066501. 25978530

[ref53] Zhou C.; Chen C.; Hu P.; Wang H. Topology-Determined Structural Genes Enable Data-Driven Discovery and Intelligent Design of Potential Metal Oxides for Inert C–H Bond Activation. J. Am. Chem. Soc. 2023, 145 (40), 21897–21903. 10.1021/jacs.3c06166. 37766450

[ref54] Grimme S.; Antony J.; Ehrlich S.; Krieg H. A consistent and accurate ab initio parametrization of density functional dispersion correction (DFT-D) for the 94 elements H-Pu. J. Chem. Phys. 2010, 132, 154104 10.1063/1.3382344. 20423165

[ref55] Tereshchuk P.; Da Silva J. L. F. Ethanol and Water Adsorption on Close-Packed 3d, 4d, and 5d Transition-Metal Surfaces: A Density Functional Theory Investigation with van der Waals Correction. J. Phys. Chem. C 2012, 116, 24695–24705. 10.1021/jp308870d. 27269477

[ref56] Shen J.; Chen C.; Song L.; Zhong L.; Shan K.; Guo Y.; Zhan W.; Guo Y.; Wang H.; Wang L. Understanding the Impact of Hydroxyls on Synthesizing the Bimetallic Alloy Pt_3_Co on Al_2_O_3_ for CO-PROX. Fuel 2025, 379, 133066 10.1016/j.fuel.2024.133066.

[ref57] Ma Y.; Chen C.; Jiang Y.; Wei X.; Liu Y.; Liao H.; Wang H.; Dai S.; An P.; Hou Z. Ruthenium Single-Atom Anchored in Polyoxometalate-Ionic Liquids for N-Formylation of Amines with CO_2_ and H_2_. ACS Catal. 2023, 13, 10295–10308. 10.1021/acscatal.3c02336.

[ref58] Takimoto D.; Toma S.; Suda Y.; Shirokura T.; Tokura Y.; Fukuda K.; Matsumoto M.; Imai H.; Sugimoto W. Platinum nanosheets synthesized via topotactic reduction of single-layer platinum oxide nanosheets for electrocatalysis. Nat. Commun. 2023, 14, 19 10.1038/s41467-022-35616-4. 36624103 PMC9829898

[ref59] Wang Y.; Zhang M.; Kang Z.; Shi L.; Shen Y.; Tian B.; Zou Y.; Chen H.; Zou X. Nano-metal diborides-supported anode catalyst with strongly coupled TaO_x_/IrO_2_ catalytic layer for low-iridium-loading proton exchange membrane electrolyzer. Nat. Commun. 2023, 14, 5119 10.1038/s41467-023-40912-8. 37612274 PMC10447464

[ref60] Luches P.; Pagliuca F.; Valeri S.; Illas F.; Preda G.; Pacchioni G. Nature of Ag Islands and Nanoparticles on the CeO_2_(111) Surface. J. Phys. Chem. C 2012, 116, 1122–1132. 10.1021/jp210241c.

[ref61] Pham T. L. M.; Nachimuthu S.; Kuo J.-L.; Jiang J.-C. A DFT study of ethane activation on IrO_2_(110) surface by precursor-mediated mechanism. Appl. Catal., A 2017, 541, 8–14. 10.1016/j.apcata.2017.04.018.

[ref62] Cai J.; Wei L.; Liu J.; Xue C.; Chen Z.; Hu Y.; Zang Y.; Wang M.; Shi W.; Qin T.; et al. Two-dimensional crystalline platinum oxide. Nat. Mater. 2024, 23, 1654–1663. 10.1038/s41563-024-02002-y. 39300286 PMC11599049

[ref63] Wang X.; Qin H.; Chen Y.; Hu J. Sensing Mechanism of SnO_2_(110) Surface to CO: Density Functional Theory Calculations. J. Phys. Chem. C 2014, 118, 28548–28561. 10.1021/jp501880r.

[ref64] Morais L. H.; López-Castillo A.; Andres J. Unraveling the mechanism of CH_3_CH_2_OH dehydrogenation on m-ZrO_2_(111) surface, Au_13_ cluster, and Au_13_ cluster/m-ZrO_2_(111) surface: A DFT and microkinetic modeling study. Appl. Surf. Sci. 2025, 680, 161418 10.1016/j.apsusc.2024.161418.

[ref65] Zhai G.; Cai L.; Ma J.; Chen Y.; Liu Z.; Si S.; Duan D.; Sang S.; Li J.; Wang X.; et al. Highly efficient, selective, and stable photocatalytic methane coupling to ethane enabled by lattice oxygen looping. Sci. Adv. 2024, 10, eado4390 10.1126/sciadv.ado4390. 38941471 PMC11637002

[ref66] Maenetja K. P.; Ngoepe P. E. First Principles Study of Oxygen Adsorption on Li-MO_2_ (M = Mn, Ti and V) (110) Surface. J. Electrochem. Soc. 2021, 168, 070556 10.1149/1945-7111/ac1640.

[ref67] Wang W.; Zhu J.; Huang Q.; Zhu L.; Wang D.; Li W.; Yu W. DFT Exploration of Metal Ion–Ligand Binding: Toward Rational Design of Chelating Agent in Semiconductor Manufacturing. Molecules 2024, 29 (2), 308 10.3390/molecules29020308. 38257221 PMC10819218

[ref68] Qi Z.; Chen L.; Zhang S.; Su J.; Somorjai G. A. Mechanism of Methanol Decomposition over Single-Site Pt_1_/CeO_2_ Catalyst: A DRIFTS Study. J. Am. Chem. Soc. 2021, 143, 60–64. 10.1021/jacs.0c10728. 33356211

[ref69] Carchini G.; López N. Adsorption of small mono- and poly-alcohols on rutile TiO_2_: a density functional theory study. Phys. Chem. Chem. Phys. 2014, 16, 14750–14760. 10.1039/c4cp01546k. 24919422

[ref70] Balajka J.; Hines M. A.; DeBenedetti W. J. I.; Komora M.; Pavelec J.; Schmid M.; Diebold U. High-affinity adsorption leads to molecularly ordered interfaces on TiO2 in air and solution. Science 2018, 361 (6404), 786–789. 10.1126/science.aat6752. 30139869

[ref71] D́Alessandro O.; Pintos D. G.; Juan A.; Irigoyen B.; Sambeth J. A DFT study of phenol adsorption on a low doping Mn–Ce composite oxide model. Appl. Surf. Sci. 2015, 359, 14–20. 10.1016/j.apsusc.2015.09.266.

[ref72] Zhu Q.; Shao Z.; Wang X.; Wang S.; Hou X.; Liang N.; Shi J.; Wu X.; Wu X.; Wang C. J. A. S. C.; et al. Computational Screening of Asymmetric Dual Sites by Bader Charge Variation Facilitates C–C Coupling for CO_2_ Photoreduction to C_2_H_4_. J. Phys. Chem. C 2024, 12, 17336–17346. 10.1021/acssuschemeng.4c07482.

[ref73] Xue M.; Nakayama M.; Liu P.; White M. G. Electronic interactions of size-selected oxide clusters on metallic and thin film oxide supports. J. Phys. Chem. C 2017, 121, 22234–22247. 10.1021/acs.jpcc.7b07889.

[ref74] Xie L.; Wang J.; Wang K.; He Z.; Liang J.; Lin Z.; Wang T.; Cao R.; Yang F.; Cai Z. J. A. C.; et al. Modulating the Bader Charge Transfer in Single p-Block Atoms Doped Pd Metallene for Enhanced Oxygen Reduction Electrocatalysis. Angew. Chem., Int. Ed. 2024, 136, e202407658 10.1002/ange.202407658. 38982589

[ref75] Zhou M.; Wang H. J. J. A. Optimally selecting photo-and electrocatalysis to facilitate CH_4_ activation on TiO_2_ (110) surface: localized photoexcitation versus global electric-field polarization. JACS Au 2022, 2, 188–196. 10.1021/jacsau.1c00466. 35098235 PMC8790734

[ref76] Gleiter R.; Haberhauer G. Aromaticity and Other Conjugation Effects; John Wiley & Sons, 2012.

[ref77] Clayden J.; Greeves N.; Warren S. Organic Chemistry; Oxford University Press: USA, 2012.

[ref78] Lazar P.; Karlický F.; Jurečka P.; Kocman M.; Otyepková E.; Šafářová K.; Otyepka M. Adsorption of Small Organic Molecules on Graphene. J. Am. Chem. Soc. 2013, 135, 6372–6377. 10.1021/ja403162r. 23570612

[ref79] Wellendorff J.; Silbaugh T. L.; Garcia-Pintos D.; Nørskov J. K.; Bligaard T.; Studt F.; Campbell C. T. A benchmark database for adsorption bond energies to transition metal surfaces and comparison to selected DFT functionals. Surf. Sci. 2015, 640, 36–44. 10.1016/j.susc.2015.03.023.

