# Supplementary material for: Descriptor-Driven Prediction of Adsorption Energy of Oxygenates on Metal Dioxide Surfaces
Source: J Phys Chem C Nanomater Interfaces. 2025 Mar 25;129(13):6245–53. doi: 10.1021/acs.jpcc.5c00005 (PMC11973912; doi:10.1021/acs.jpcc.5c00005)
Supplement: Supplementary file 1 — jp5c00005_si_001.pdf [file jp5c00005_si_001.pdf]

Supplementary Information

Descriptor-Driven Prediction of Adsorption Energy of Oxygenates on Metal Dioxide Surfaces

Chen Chen[ab], Zhihui Li[b], Jia Yang[c]*, Haifeng Wang[a]*, De Chen[b]*

[a]Key Laboratory for Advanced Materials, Centre for Computational Chemistry and Research Institute of Industrial Catalysis, East China University of Science and Technology; Shanghai, 200237, China.

[b]Department of Chemical Engineering, Norwegian University of Science and Technology; Trondheim, 7034, Norway.

[c]The College of Smart Energy, Shanghai Jiao Tong University; Shanghai, 200237, China.

*Corresponding author: de.chen@ntnu.no

Number of pages: 17

Number of figures: 8

Number of tables: 21

Supplementary Note 1: Supplementary calculation methods

1.1 Effect of Hubbard U values on the results.

1.1.1 Effect of Hubbard U values on electron affinity (EA):

Since HSE06 is known for its high accuracy, it has been widely used in previous studies to benchmark the reliability of DFT+U calculations.[1-3] Therefore, we compared the EA values of all MO_2 systems using three different methods: DFT (without U), DFT+U and HSE06 hybrid functional.

Table S1. The EA values of all MO_2 systems using three different methods: DFT+U, DFT (without U), and HSE06 hybrid functional. All energy values are reported in electron volts (eV).

	**DFT (without U)**	**DFT+U**	**HSE06**
**IrO_2**	0.44	0.21	0.2
**SnO_2**	0.57	0.45	0.43
**TiO_2**	1.05	0.91	0.86
**PtO_2**	1.45	1.15	1.25
**ZrO_2**	2.06	1.93	1.89
**CeO_2**	2.7	2.04	1.98

As shown in Table S1, the EA values obtained using DFT+U are in good agreement with those from HSE06, which validates the rationality of our chosen U values. However, calculations without U tend to overestimate EA values. This is because the introduction of the U correction enhances the localization of d-electrons, leading to an upward shift in the conduction band minimum (CBM) of metal dioxide. As a result, the LUMO energy level increases, causing a decrease in EA. In other words, if DFT+U is not applied, the system's attraction to additional electrons is overestimated, leading to an artificially large EA value. This confirms that the U correction effectively improves the accuracy of EA predictions and better aligns with hybrid functional results.

1.1.2 Effect of Hubbard U on adsorption energy (E_{ad}):

To further assess the impact of the U correction on adsorption energy, we selected ester adsorption as a representative case and compared the adsorption energies (E_{ad}) on six different MO_2 surfaces using DFT+U, DFT (without U), and HSE06.

As shown in Table S2, the adsorption energies calculated using DFT+U closely match those obtained from HSE06, whereas adsorption energies from calculations without U tend to be underestimated. This confirms the accuracy of our DFT+U approach.

Table S2. The adsorption energies (E_{ad}) on six different MO_2 surfaces using three different methods: DFT+U, DFT (without U), and HSE06 hybrid functional.

	**DFT (without U)**	**DFT+U**	**HSE06**
**IrO_2**	-0.75	-1.27	-1.3
**SnO_2**	-0.6	-1.01	-1
**TiO_2**	-0.5	-0.74	-0.79
**PtO_2**	-0.49	-0.66	-0.63
**ZrO_2**	-0.32	-0.39	-0.39
**CeO_2**	-0.21	-0.34	-0.36

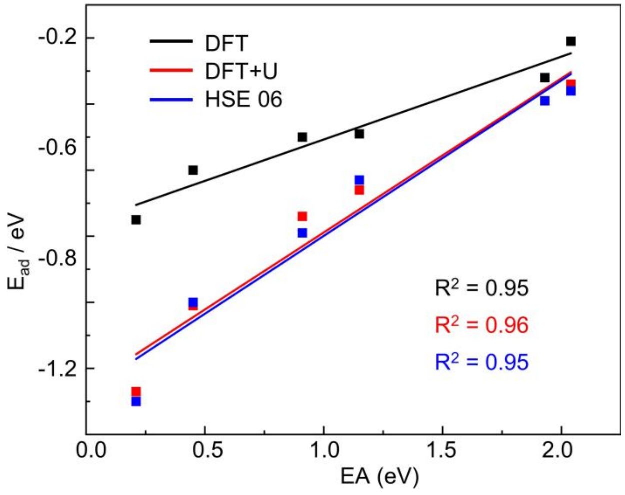

Figure S1. The linear fitting analyses of the adsorption energies obtained from different computational methods (DFT, DFT+U, and HSE06) with respect to the e_{eff} and EA.

As shown in Figure S1, we have performed linear fitting analyses of the adsorption energies obtained from different computational methods (DFT, DFT+U, and HSE06) with respect to the two descriptors, e_{eff} and EA. Despite differences in absolute adsorption energy values, the trends remain consistent across

functionals. Therefore, using a well-calibrated U value accurately describes the localization of metal d orbitals in MO_2, leading to a correct estimation of adsorption strength between MO_2 and oxygenates.

1.2 Effect of zero-point energy and entropy

To illustrate how the results of the calculations depend on the extracted zero-point energy (ΔZPE) and entropy contributions (ΔS), we used the following equations to account for the ΔZPE and ΔS:

- Initial adsorption energy (E_{ad1}): $E_{ad1} = E_{X/surf} - E_{surf} - E_X$
- Adsorption energy only with ΔZPE (E_{ad2}): $E_{ad2} = E_{X/surf} - E_{surf} - E_X + \Delta ZPE$
- Adsorption energy only with TΔS (E_{ad3}): $E_{ad3} = E_{X/surf} - E_{surf} - E_X + T\Delta S$
- Final adsorption energy including ΔZPE and ΔS (E_{ad}): $E_{ad} = E_{X/surf} - E_{surf} - E_X + \Delta ZPE + T\Delta S$.

We have also added Table S3-8 to present the adsorption energies with and without the zero-point energy and entropy contributions for various oxygenates on six different MO_2 surfaces:

Table S3. E_{ad1}, ΔZPE, E_{ad2}, TΔS, E_{ad3} and E_{ad} on IrO_2 surface.

	E_{ad1}	ΔZPE	E_{ad2}	TΔS	E_{ad3}	E_{ad}
**Ester**	-1.5	0.09	-1.41	0.14	-1.36	-1.27
**Ketone**	-1.39	0.11	-1.28	0.11	-1.28	-1.17
**Phenol**	-1.31	0.1	-1.21	0.06	-1.25	-1.15
**Acid**	-1.41	0.08	-1.33	0.22	-1.19	-1.11
**Aldehyde**	-1.25	0.09	-1.16	0.07	-1.18	-1.09
**Alcohol**	-1.18	0.07	-1.11	0.05	-1.13	-1.06
**Ether**	-0.99	0.06	-0.93	0.04	-0.95	-0.89

Table S4. E_{ad1}, ΔZPE, E_{ad2}, TΔS, E_{ad3} and E_{ad} on SnO_2 surface.

	E_{ad1}	ΔZPE	E_{ad2}	TΔS	E_{ad3}	E_{ad}
**Ester**	-1.17	0.11	-1.06	0.05	-1.12	-1.01
**Ketone**	-1.18	0.11	-1.07	0.12	-1.06	-0.95
**Phenol**	-1.13	0.16	-0.97	0.06	-1.07	-0.91
**Acid**	-1.21	0.14	-1.07	0.17	-1.04	-0.90
**Aldehyde**	-1.12	0.1	-1.02	0.15	-0.97	-0.87

**Alcohol**	-0.99	0.12	-0.87	0.04	-0.95	-0.83
**Ether**	-0.8	0.08	-0.72	0.06	-0.74	-0.66

Table S5. E_{ad1}, ΔZPE, E_{ad2}, TΔS, E_{ad3} and E_{ad} on TiO_2 surface.

	E_{ad1}	ΔZPE	E_{ad2}	TΔS	E_{ad3}	E_{ad}
**Ester**	-0.86	0.07	-0.79	0.05	-0.81	-0.74
**Ketone**	-0.84	0.09	-0.75	0.07	-0.77	-0.68
**Phenol**	-0.89	0.15	-0.74	0.08	-0.81	-0.66
**Acid**	-0.98	0.14	-0.84	0.22	-0.76	-0.62
**Aldehyde**	-0.81	0.08	-0.73	0.13	-0.68	-0.60
**Alcohol**	-0.75	0.11	-0.64	0.06	-0.69	-0.58
**Ether**	-0.52	0.06	-0.46	0.07	-0.45	-0.39

Table S6. E_{ad1}, ΔZPE, E_{ad2}, TΔS, E_{ad3} and E_{ad} on PtO_2 surface.

	E_{ad1}	ΔZPE	E_{ad2}	TΔS	E_{ad3}	E_{ad}
**Ester**	-0.77	0.07	-0.7	0.04	-0.73	-0.66
**Ketone**	-0.73	0.09	-0.64	0.06	-0.67	-0.58
**Phenol**	-0.71	0.12	-0.59	0.02	-0.69	-0.57
**Acid**	-0.84	0.11	-0.73	0.2	-0.64	-0.53
**Aldehyde**	-0.63	0.08	-0.55	0.06	-0.57	-0.49
**Alcohol**	-0.77	0.1	-0.67	0.19	-0.58	-0.48
**Ether**	-0.46	0.05	-0.41	0.11	-0.35	-0.30

Table S7. E_{ad1}, ΔZPE, E_{ad2}, TΔS, E_{ad3} and E_{ad} on ZrO_2 surface.

	E_{ad1}	ΔZPE	E_{ad2}	TΔS	E_{ad3}	E_{ad}
**Ester**	-0.6	0.18	-0.42	0.03	-0.57	-0.39
**Ketone**	-0.54	0.18	-0.36	0.02	-0.52	-0.34
**Phenol**	-0.65	0.26	-0.39	0.08	-0.57	-0.31
**Acid**	-0.67	0.24	-0.43	0.15	-0.52	-0.28

**Aldehyde**	-0.44	0.17	-0.27	0.03	-0.41	-0.24
**Alcohol**	-0.56	0.2	-0.36	0.14	-0.42	-0.22
**Ether**	-0.28	0.12	-0.16	0.12	-0.16	-0.04

Table S8. E_{ad1}, ΔZPE, E_{ad2}, TΔS, E_{ad3} and E_{ad} on CeO_2 surface.

	E_{ad1}	ΔZPE	E_{ad2}	TΔS	E_{ad3}	E_{ad}
**Ester**	-0.62	0.25	-0.37	0.03	-0.59	-0.34
**Ketone**	-0.56	0.24	-0.32	0.02	-0.54	-0.30
**Phenol**	-0.63	0.32	-0.31	0.02	-0.61	-0.29
**Acid**	-0.7	0.28	-0.42	0.17	-0.53	-0.25
**Aldehyde**	-0.5	0.22	-0.28	0.06	-0.44	-0.22
**Alcohol**	-0.55	0.27	-0.28	0.08	-0.47	-0.20
**Ether**	-0.25	0.16	-0.09	0.07	-0.18	-0.02

Using the IrO_2 surface as an example, we have performed the fitting analysis of E_{ad1}, E_{ad2}, E_{ad3} and E_{ad} with respect to e_{eff}. As shown in Figure S2, the fitting relationships between E_{ad3} and E_{ad} with e_{eff} are very good, indicating a strong correlation. However, the fitting relationships for E_{ad1} and E_{ad2} are not as good.

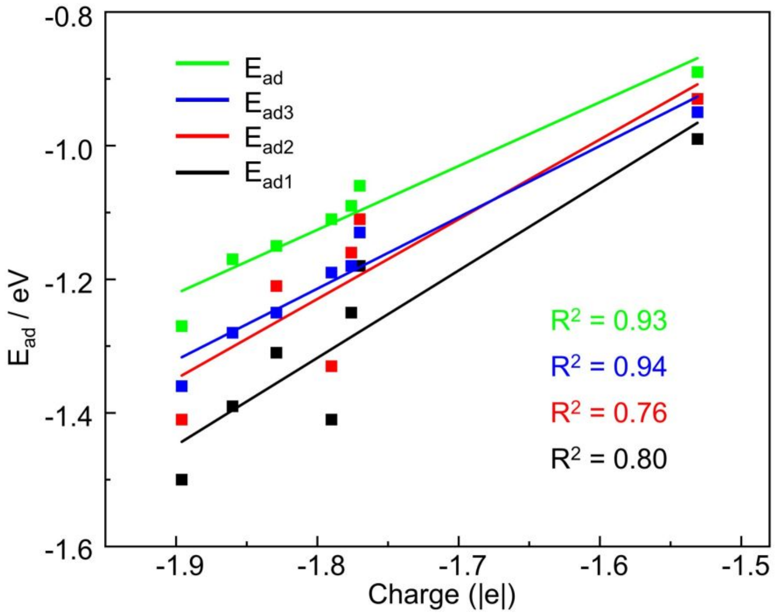

Figure S2. The fitting relationships between E_{ad1}, E_{ad2}, E_{ad3} and E_{ad} with respect to e_{eff}.

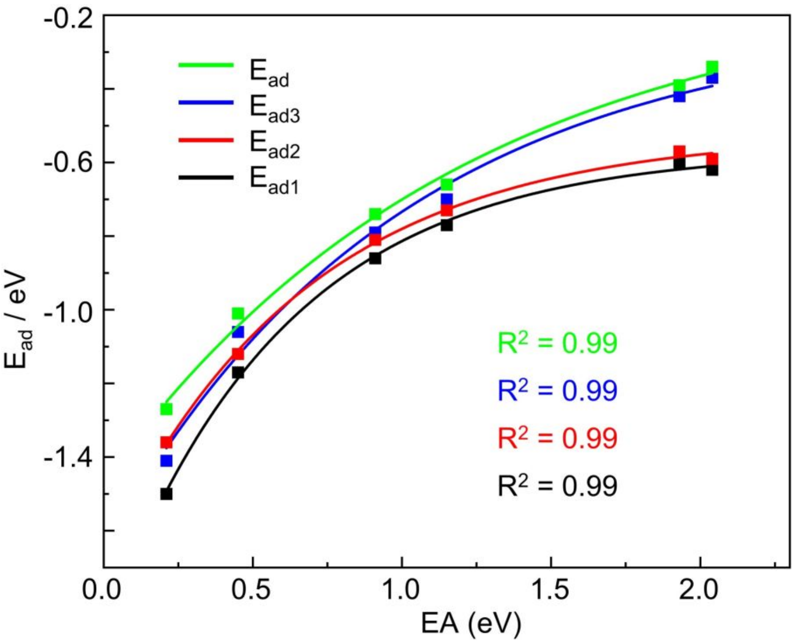

Figure S3. The fitting relationships between E_{ad1}, E_{ad2}, E_{ad3} and E_{ad} with respect to EA.

Furthermore, using esters as a representative case, we have analyzed the adsorption energy variations (E_{ad1}, E_{ad2}, E_{ad3} and E_{ad}) across different MO_2 surfaces with respect to EA. As illustrated in Figure S3, while all four adsorption energy calculations exhibit high R^2 values, the curvature of the fitting curves for E_{ad1} and E_{ad2} differs significantly from that of E_{ad3} and E_{ad}. This indicates that the accuracy of adsorption energy predictions is substantially affected when ΔZPE and ΔS are not fully considered.

These suggest that although both the ΔZPE and ΔS have an impact on the absolute value of the adsorption energy, the ΔZPE does not significantly affect the adsorption trend, while the ΔS plays a more pronounced role. Therefore, considering both ΔZPE and ΔS is crucial for accurately calculating the adsorption energy.

Supplementary Note 2: Supplementary Figures

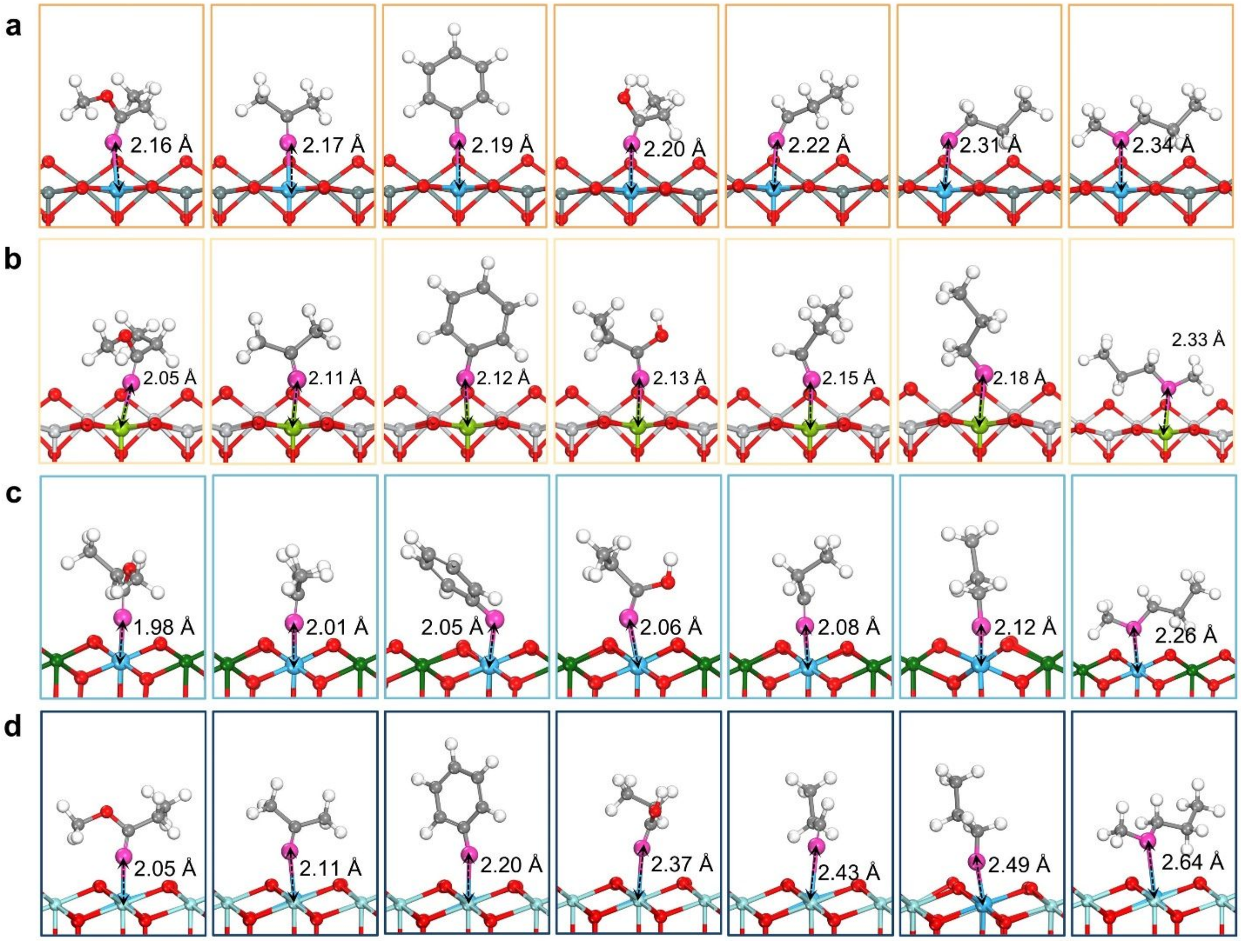

Figure S4. Adsorption structures of oxygenates on the surfaces of rutile-SnO_2(110), rutile-TiO_2(110), tetragonal-PtO_2(111), tetragonal-ZrO_2(111). Black markers denote bond lengths.

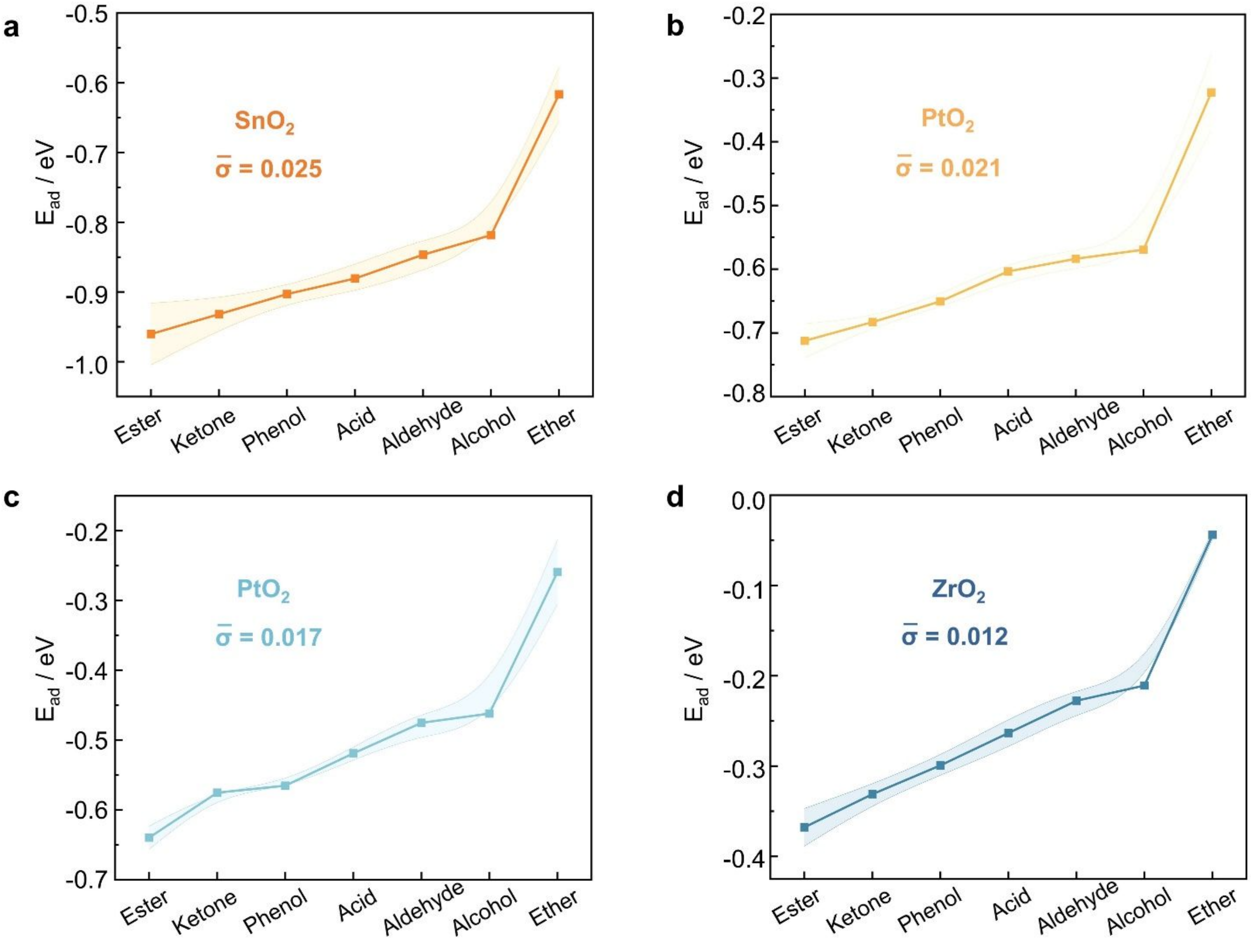

Figure S5. Error bar chart depicting the adsorption energies of oxygenates with varying carbon chain lengths (C3, C4, and C6) on the surfaces of rutile-SnO_2(110), rutile-TiO_2(110), tetragonal-PtO_2(111), tetragonal-ZrO_2(111). In the case of phenol, the C3, C4, and C6 designations refer to the addition of alkyl groups ($–C_3H_7$, $–C_4H_9$, and $–C_6H_{13}$) to the benzene ring, corresponding to phenol derivatives with different carbon chain lengths.

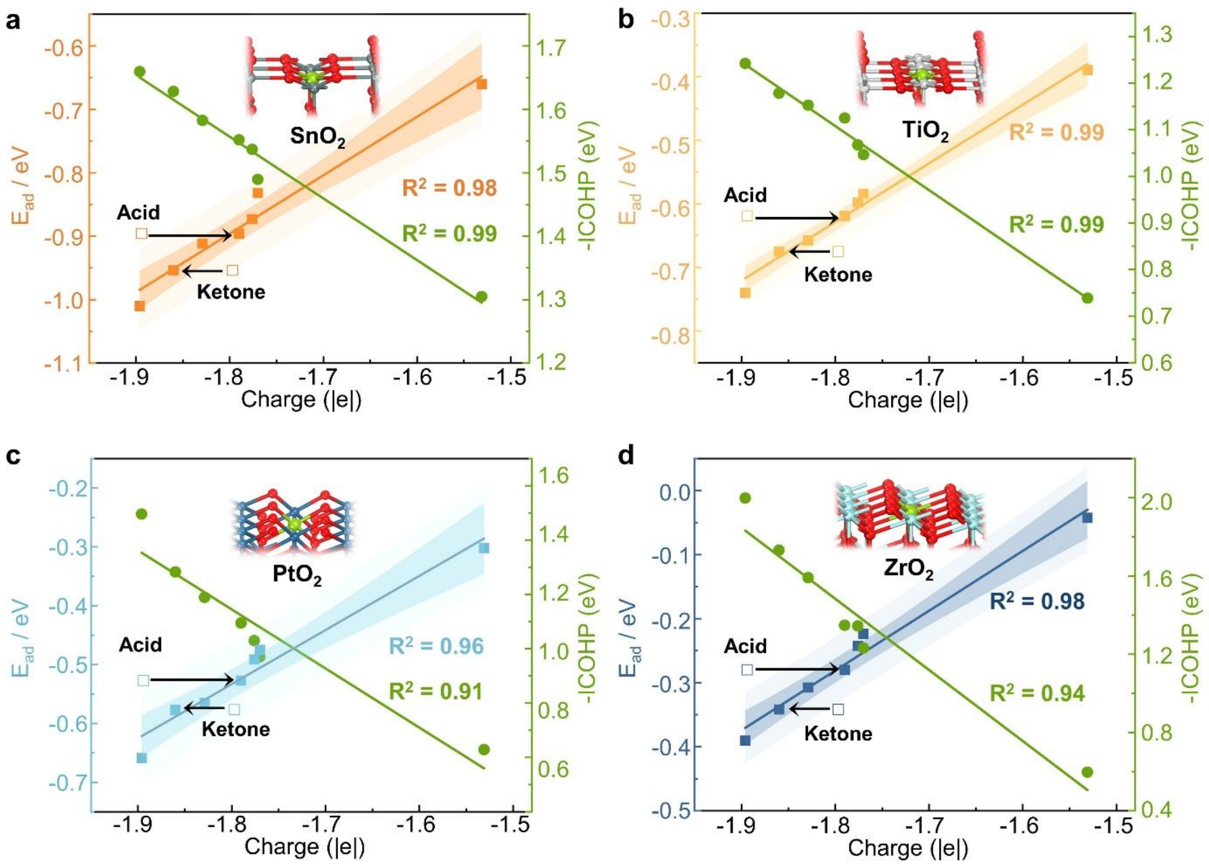

Figure S6. Correlation between the charges of oxygen atoms in oxygenates and the adsorption energies on the surfaces of rutile-SnO_2(110), rutile-TiO_2(110), tetragonal-PtO_2(111), tetragonal-ZrO_2(111), as well as M-O bond energies (-ICOHP). The yellow and blue lines represent the best-fit regression models for the adsorption energies on these surfaces. The green line shows the correlation between the effective charge and M-O bond energies. The shaded regions around the lines represent the 95% confidence intervals, indicating the uncertainty in the predicted mean values. The outer shaded regions represent the 95% prediction intervals, showing the expected range for future observations with 95% confidence.

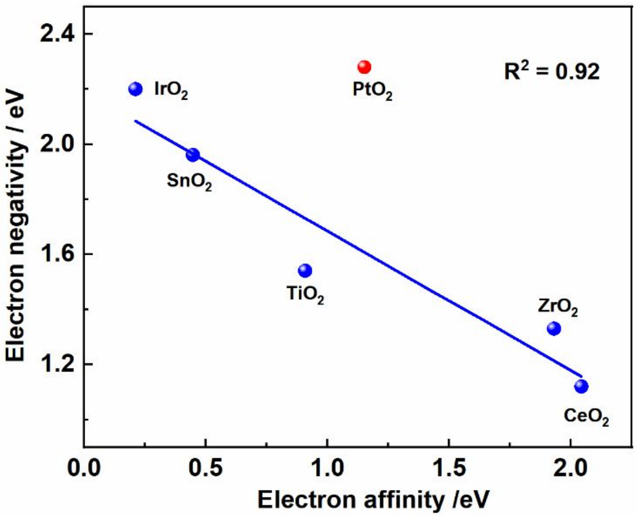

Figure S7. Correlation between electron affinity energy and electron negativity of metallic elements in MO_2.

With the exception of PtO_2, the EA of the metal atoms on the MO_2 surface correlate well with the electron negativity of their elements. This is because the EA of metal dioxide are not only dependent on the electronic properties of the constituent metals, but are also influenced by the crystal structure of the oxide, the nature of the oxygen element, and the bonding interactions between the metal and oxygen atoms. Although elemental Pt has a high electron affinity energy, the surface of PtO_2 is a sawtooth structure, which also affects the electron trapping ability (or EA) of the atoms on its surface. Therefore, PtO_2 does not fulfill the scaling relationship between EA and electron negativity.

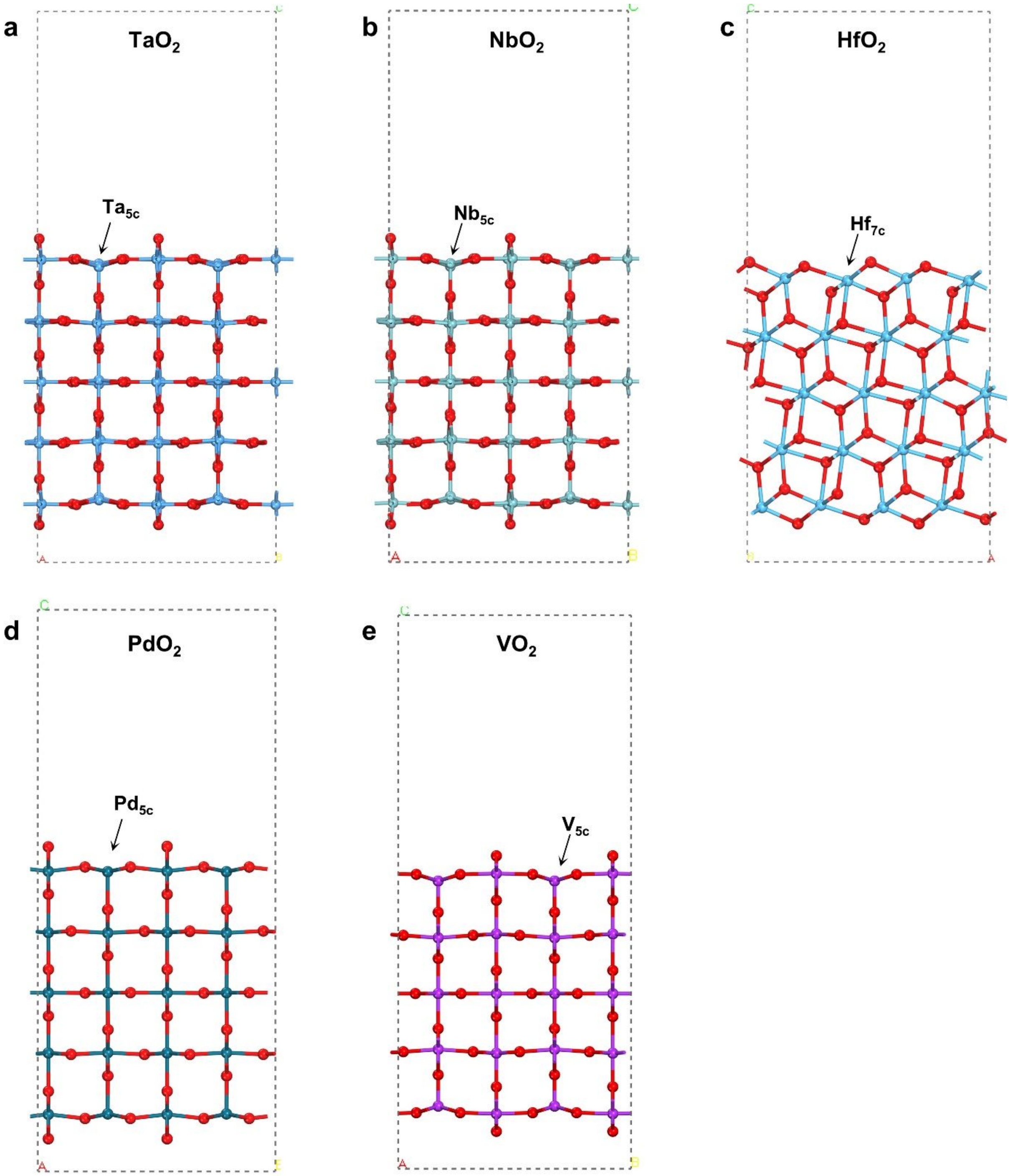

Figure S8. Surface structure of rutile-TaO_2(100), rutile-NbO_2(100), tetragonal-HfO_2(-101), rutile-PdO_2(100) and rutile-VO_2(100).

Supplementary Note 3: Supplementary Tables

Table S9. Adsorption energies of oxygenates with C3, C4, and C6 on rutile-IrO_2(110).

	C3	C4	C6
**Ester**	-1.27	-1.24	-1.21
**Ketone**	-1.17	-1.14	-1.12
**Phenol**	-1.15	-1.08	-1.05
**Acid**	-1.11	-1.07	-1.04
**Aldehyde**	-1.09	-1.08	-1.07
**Alcohol**	-1.06	-1.07	-1.05
**Ether**	-0.89	-0.81	-0.77

Table S10. Adsorption energies of oxygenates with C3, C4, and C6 on rutile-SnO_2(110).

	C3	C4	C6
**Ester**	-1.01	-0.95	-0.92
**Ketone**	-0.95	-0.93	-0.91
**Phenol**	-0.91	-0.91	-0.89
**Acid**	-0.90	-0.89	-0.86
**Aldehyde**	-0.87	-0.84	-0.83
**Alcohol**	-0.83	-0.82	-0.81
**Ether**	-0.66	-0.60	-0.59

Table S11. Adsorption energies of oxygenates with C3, C4, and C6 on rutile-TiO_2(110).

	C3	C4	C6
**Ester**	-0.74	-0.71	-0.69
**Ketone**	-0.68	-0.69	-0.68
**Phenol**	-0.66	-0.65	-0.64
**Acid**	-0.62	-0.60	-0.59
**Aldehyde**	-0.60	-0.58	-0.57
**Alcohol**	-0.58	-0.57	-0.55

Ether	-0.39	-0.30	-0.28

Table S12. Adsorption energies of oxygenates with C3, C4, and C6 on tetragonal-PtO_2(111).

	**C3**	**C4**	**C6**
**Ester**	-0.66	-0.63	-0.63
**Ketone**	-0.58	-0.58	-0.57
**Phenol**	-0.57	-0.57	-0.56
**Acid**	-0.53	-0.52	-0.51
**Aldehyde**	-0.49	-0.48	-0.46
**Alcohol**	-0.48	-0.47	-0.44
**Ether**	-0.30	-0.27	-0.21

Table S13. Adsorption energies of oxygenates with C3, C4, and C6 on tetragonal-ZrO_2(111).

	**C3**	**C4**	**C6**
**Ester**	-0.39	-0.36	-0.35
**Ketone**	-0.34	-0.33	-0.32
**Phenol**	-0.31	-0.30	-0.29
**Acid**	-0.28	-0.26	-0.25
**Aldehyde**	-0.24	-0.22	-0.22
**Alcohol**	-0.22	-0.20	-0.20
**Ether**	-0.04	-0.04	-0.05

Table S14. Adsorption energies of oxygenates with C3, C4, and C6 on tetragonal-CeO_2(111).

	**C3**	**C4**	**C6**
**Ester**	-0.34	-0.33	-0.29
**Ketone**	-0.30	-0.30	-0.28
**Phenol**	-0.29	-0.29	-0.28
**Acid**	-0.25	-0.24	-0.23
**Aldehyde**	-0.22	-0.22	-0.21
**Alcohol**	-0.20	-0.21	-0.19

**Ether**	-0.02	-0.04	-0.01

Table S15. Adsorption energies of oxygenates on the surfaces of rutile-IrO_2(110), rutile-SnO_2(110), rutile-TiO_2(110), tetragonal-PtO_2(111), tetragonal-ZrO_2(111), and tetragonal-CeO_2(111).

	**IrO_2**	**SnO_2**	**TiO_2**	**PtO_2**	**ZrO_2**	**CeO_2**
**Ester**	-1.27	-1.01	-0.74	-0.66	-0.39	-0.34
**Ketone**	-1.17	-0.95	-0.68	-0.58	-0.34	-0.30
**Phenol**	-1.15	-0.91	-0.66	-0.57	-0.31	-0.29
**Acid**	-1.11	-0.90	-0.62	-0.53	-0.28	-0.25
**Aldehyde**	-1.09	-0.87	-0.60	-0.49	-0.24	-0.22
**Alcohol**	-1.06	-0.83	-0.58	-0.48	-0.22	-0.20
**Ether**	-0.89	-0.66	-0.39	-0.30	-0.04	-0.02

Table S16. Bond energies (-ICOHP) of M-O on the rutile-IrO_2(110), rutile-SnO_2(110), rutile-TiO_2(110), tetragonal-PtO_2(111), tetragonal-ZrO_2(111), and tetragonal-CeO_2(111) surfaces.

	**IrO_2**	**SnO_2**	**TiO_2**	**PtO_2**	**ZrO_2**	**CeO_2**
**Ester**	1.769	1.660	1.242	1.496	1.998	1.317
**Ketone**	1.521	1.628	1.178	1.283	1.732	1.283
**Phenol**	1.386	1.582	1.153	1.189	1.591	1.211
**Acid**	1.293	1.552	1.125	1.095	1.348	1.175
**Aldehyde**	1.273	1.537	1.067	1.030	1.345	1.153
**Alcohol**	1.100	1.490	1.046	0.970	1.231	1.138
**Ether**	0.450	1.305	0.739	0.629	0.597	1.027

Table S17. Bond lengths of M-O on the rutile-IrO_2(110), rutile-SnO_2(110), rutile-TiO_2(110), tetragonal-PtO_2(111), tetragonal-ZrO_2(111), and tetragonal-CeO_2(111) surfaces.

	**IrO_2**	**SnO_2**	**TiO_2**	**PtO_2**	**ZrO_2**	**CeO_2**
**Ester**	1.949	2.160	2.048	1.984	2.053	2.384
**Ketone**	1.990	2.166	2.108	2.010	2.113	2.415
**Phenol**	2.014	2.191	2.120	2.047	2.195	2.502
**Acid**	2.044	2.202	2.124	2.055	2.373	2.560
**Aldehyde**	2.072	2.220	2.146	2.083	2.425	2.569
**Alcohol**	2.107	2.280	2.181	2.117	2.489	2.593

Ether	2.286	2.435	2.330	2.259	2.644	2.765

Table S18. A concise database of electron affinity (EA) for MO_2 and the effective charge (e_{eff}) of oxygen atoms in oxygenates.

Oxygenates	e_{eff}	MO_2	EA
Ester	-1.896	IrO_2	0.211
Ketone	-1.797	SnO_2	0.448
Phenol	-1.829	TiO_2	0.910
Acid	-1.894	PtO_2	1.152
Aldehyde	-1.776	ZrO_2	1.932
Alcohol	-1.770	CeO_2	2.044
Ether	-1.531	PdO_2	1.440
-	-	VO_2	1.231
-	-	NbO_2	0.982
-	-	HfO_2	0.503
-	-	TaO_2	-0.856

Table S19. The proportional relationship between E_{ad} and e_{eff} is given by $E_{ad} = k_x e_{eff} + b_x$. The table presents the values of k_x (slope), b_x (intercept), and R^2 (goodness-of-fit).

	IrO_2	SnO_2	TiO_2	PtO_2	ZrO_2	CeO_2
k_x	0.955	0.922	0.923	0.920	0.933	0.883
b_x	0.593	0.764	1.033	1.123	1.399	1.339
R^2	0.939	0.968	0.986	0.958	0.976	0.983

Table S20. The proportional relationship between E_{ad} and EA is given by $E_{ad} = A_x e^{-\mathbf{EA}} + c_x$. The table presents the values of A_x, c_x, and R^2.

	Ester	Ketone	Phenol	Acid	Aldehyde	Alcohol	Ether
A_x	-1.352	-1.287	-1.281	-1.285	-1.298	-1.304	-1.306
c_x	-0.206	-0.140	-0.117	-0.084	-0.057	-0.032	0.146
R^2	0.999	0.999	0.996	0.999	0.997	0.998	0.999

Table S21. E_{ad} of oxygenates on the surface of rutile-PdO_2(100), rutile-VO_2(100), rutile-TaO_2(100), tetragonal-HfO_2(-101) and rutile-NbO_2(100), including E_{ad} obtained through DFT calculations ($E_{ad\text{-}DFT}$) and predicted ($E_{ad\text{-}Pr}$) based on the Eq.5.

	**PdO_2**		**VO_2**		**NbO_2**		**HfO_2**		**TaO_2**	
	**$E_{ad\text{-}Pr}$**	**$E_{ad\text{-}DFT}$**	**$E_{ad\text{-}Pr}$**	**$E_{ad\text{-}DFT}$**	**$E_{ad\text{-}Pr}$**	**$E_{ad\text{-}DFT}$**	**$E_{ad\text{-}Pr}$**	**$E_{ad\text{-}DFT}$**	**$E_{ad\text{-}Pr}$**	**$E_{ad\text{-}DFT}$**
**Ester**	-0.58	-0.60	-0.66	-0.65	-0.77	-0.70	-0.96	-1.03	-1.52	-1.60
**Ketone**	-0.54	-0.54	-0.63	-0.60	-0.73	-0.63	-0.93	-0.94	-1.49	-1.57
**Phenol**	-0.52	-0.51	-0.60	-0.57	-0.70	-0.60	-0.90	-0.92	-1.46	-1.59
**Acid**	-0.48	-0.43	-0.57	-0.52	-0.67	-0.56	-0.86	-0.88	-1.42	-1.54
**Aldehyde**	-0.47	-0.39	-0.55	-0.46	-0.65	-0.52	-0.85	-0.86	-1.41	-1.49
**Alcohol**	-0.46	-0.37	-0.55	-0.47	-0.65	-0.50	-0.85	-0.83	-1.40	-1.47
**Ether**	-0.24	-0.17	-0.33	-0.29	-0.43	-0.35	-0.63	-0.67	-1.18	-1.28

References

(1) Wang, D.; Sheng, T.; Chen, J.; Wang, H.-F.; Hu, P., Identifying the key obstacle in photocatalytic oxygen evolution on rutile TiO_2. *Nat. Catal.* **2018,** *1*, 291-299.
(2) Zhang, J.; Peng, C.; Wang, H.; Hu, P., Identifying the Role of Photogenerated Holes in Photocatalytic Methanol Dissociation on Rutile TiO_2(110). *ACS Catal.* **2017,** *7*, 2374-2380.
(3) Ren, G.; Zhou, M.; Wang, H., Weakened Interfacial Hydrogen Bond Connectivity Drives Selective Photocatalytic Water Oxidation toward H_2O_2 at Water/Brookite-TiO_2 Interface. *J. Am. Chem. Soc.* **2024,** *146*, 6084-6093.
